# Evaluation of vaccination strategies for SIR epidemics on random networks incorporating household structure

**DOI:** 10.1007/s00285-017-1139-0

**Published:** 2017-06-20

**Authors:** Frank Ball, David Sirl

**Affiliations:** School of Mathematical Sciences, University of Nottingham, University Park, Nottingham, NG7 2RD UK

**Keywords:** Branching process, Configuration model, Epidemic process, Final size, Random graph, Threshold behaviour, Vaccination, Primary 92D30 (Epidemiology), Secondary 60J85 (Applications of BPs), 05C80 (Random graphs)

## Abstract

This paper is concerned with the analysis of vaccination strategies in a stochastic susceptible $\rightarrow$ infected $\rightarrow$ removed model for the spread of an epidemic amongst a population of individuals with a random network of social contacts that is also partitioned into households. Under various vaccine action models, we consider both household-based vaccination schemes, in which the way in which individuals are chosen for vaccination depends on the size of the households in which they reside, and acquaintance vaccination, which targets individuals of high degree in the social network. For both types of vaccination scheme, assuming a large population with few initial infectives, we derive a threshold parameter which determines whether or not a large outbreak can occur and also the probability of a large outbreak and the fraction of the population infected by a large outbreak. The performance of these schemes is studied numerically, focusing on the influence of the household size distribution and the degree distribution of the social network. We find that acquaintance vaccination can significantly outperform the best household-based scheme if the degree distribution of the social network is heavy-tailed. For household-based schemes, when the vaccine coverage is insufficient to prevent a major outbreak and the vaccine is imperfect, we find situations in which both the probability and size of a major outbreak under the scheme which minimises the threshold parameter are *larger* than in the scheme which maximises the threshold parameter.

## Introduction and description of results

Mathematical models for the spread of infectious disease have much to offer in terms of understanding past outbreaks, predicting likely behaviours of future outbreaks and predicting the effect of interventions or mitigating strategies. In the last decade or two there has been considerable interest and work on network epidemic models. These involve supplanting the traditional assumption of homogeneous mixing of homogeneous individuals with some random graph structure, with specific interest in being able to control the degree distribution, reflecting the varying numbers of people with which different individuals tend to interact. Other structures, for example including households and stratification of populations, have been studied for longer (Bartoszyński 1972; Ball et al. 1997; Watson 1972; Scalia-Tomba 1986); but structures with a ‘social network’ type of interpretation start around the turn of the millenium with the works of Andersson (1997, 1998), Diekmann et al. (1998) and Newman (2002). In this and most other papers in the field we typically have in mind an infection spreading through a human population. However, much the same ideas apply to mathematical models of a variety of other motivating applications, such as the spread of rumours or information through human populations, infection or information spread through a population of other animals and virus spread through a network of computers.

In this paper we build on the model of Ball et al. (2009, 2010) which includes household and network structure to include vaccination, with some emphasis on so-called acquaintance vaccination (Cohen et al. 2003; Britton et al. 2007) as elucidated in Ball and Sirl (2013) in a model without household structure. In the model of Ball et al. (2010), a population of fixed size is given social network structure via the configuration model random graph (see e.g. Bollobás 1980; Newman et al. 2001; Newman 2002) and the population is also partitioned into households (see e.g. Ball et al. 1997). A stochastic Susceptible $\rightarrow$ Infective $\rightarrow$ Removed (SIR) epidemic model is then defined on this population structure. A first quantity of interest in this model is the final size, which is the (random) number of initial susceptibles that are infected at some point during the epidemic. In line with much of modern stochastic epidemic theory, one can use branching process approximations to prove a threshold theorem (valid in the large population limit) which determines whether the infection will necessarily die out relatively quickly, resulting in a small final size, or whether it is possible for the epidemic to take off and infect a substantial fraction of the population. These methods also yield approximations for the probability that a supercritical epidemic will take off and using closely related methods one can also study final size properties of such a large outbreak.

This paper provides tools for studying the effect of introducing vaccination into this model. *Households-based* vaccination schemes are those that can be described in terms of the distribution of the number of vaccinated individuals in households of size *n*, for every household size *n* in the population. This includes as special cases the situation when we vaccinate individuals who are chosen uniformly at random from the population (the distributions are binomial) and vaccinating households at random (the distributions are concentrated at 0 and *n*). Optimal schemes in this context often resemble the equalising strategy (Ball et al. 1997, Sect. 5.2) where one vaccinates preferentially in larger households, there being more of a herd immunity effect available to exploit in those larger households on top of the direct protection of vaccinated individuals. *Acquaintance vaccination* schemes exploit the heterogeneity of individuals’ connectivities in a network to preferentially target better-connected individuals for vaccination. Instead of vaccinating individuals chosen from the population in some way, one samples individuals and then vaccinates their friends—their acquaintances in the network. This exploits the so-called friendship paradox: the observation that, for most people, their friends on average have more friends than they do (Feld 1991).

Our main theoretical results are the calculation of asymptotic final size quantities, via appropriate branching process approximations, when we include vaccination in Ball et al.’s (2010) household-network model. This extends Becker and Starczak’s (1997) and Ball and Lyne’s (2002, 2006) results on the standard households model to have network-based (rather than homogeneous mixing) casual contacts. It also extends Ball and Sirl’s (2013) results on acquaintance vaccination in a population with network (but not household) structure. We also explore the model numerically and find that there can be substantial differences in the performance of the different vaccine allocation strategies.

Results like those in this paper can inform about which mechanism of spreading (household or network) can be most beneficially targeted for reducing the impact of outbreaks of disease. This seems likely to be relevant when considering, for example, how best to reduce the impact of any future Ebola outbreak in West Africa (Adams 2016) using vaccines that are currently under development. Other possible applications include pandemic influenza (Halloran et al. 2008) and smallpox (Halloran et al. 2002). For all of these diseases a salient feature is that they have markedly enhanced transmission within small social groups such as households. In each of these possible applications some further refinements of the underlying model may be valuable.

The remainder of the paper is structured as follows. In Sect. 2 we specify our models for the population structure and evolution of the epidemic, then outline the analysis of the final outcome of the epidemic and lastly introduce the model we use for the action of a vaccine on individuals who receive it. In Sect. 3 we consider the effect of households-based vaccination, including optimal households-based strategies, and in Sect. 4 we consider the effect of acquaintance vaccination. The analysis in these sections is of the same final outcome measures as in the basic model in Sect. 2. Some exploration of the behaviour of the model (mainly numerical) is presented in Sect. 5. Lastly we offer some concluding remarks in Sect. 6. Details of many of the calculations relating to Sect. 4 are given in Appendix A, while in Appendix B some known exact results for the final outcome and susceptibility sets of multitype stochastic SIR epidemics (households in our setting) are stated and applied to the asymptotic analyses of Sects. 2, 3 and 4.

## Model, threshold behaviour and vaccination

### Model

The model we consider is one for the spread of an SIR epidemic on a finite random network incorporating household structure (Ball et al. 2010). We assume that the population consists of *N* individuals and is partitioned into *m* households, of which $m_n$ are of size *n* ($n=1,2,\ldots$). Thus $m=\sum _{n=1}^{\infty } m_n$ and $N=\sum _{n=1}^{\infty } nm_n$. The network of possible global (i.e. between-household) contacts is constructed using the configuration model (with random, rather than specified, degree sequence). Thus each individual is assigned a number of ‘half-edges’ independently, according to an arbitrary but specified discrete random variable *D* having mass function $P(D=k)=p_k$ ($k=0,1,\ldots$). Then all such half-edges are paired up uniformly at random to form the edges in the graph describing the global network. If the total number of half-edges is odd, we ignore the single leftover half-edge.

Our analysis is asymptotic as the number of households $m \rightarrow \infty$. We require that, as $m \rightarrow \infty$, $m_n / m \rightarrow \rho _n$ ($n=1,2,\ldots$), where $(\rho _1, \rho _2 , \ldots$) is a proper probability distribution (i.e. $\sum _{n=1}^{\infty } \rho _n = 1$) having finite mean $\mu _H = \sum _{n=1}^{\infty } n \rho _n$. Thus $\mu _H$ is the mean household size in the limiting population. We also require that $\mu _D = E[D]$ is finite. These assumptions are sufficient for our analysis. If we make the stronger assumptions that $\sigma _D^2 = {\mathrm {var}}(D)$ and $\sum _{n=1}^{\infty } n^2 \rho _n$ are both finite, then parallel edges and self-loops, between either individuals or households, become sparse in the global network as $n \rightarrow \infty$ (Ball et al. 2010, Sect. 1.2).

The epidemic is initiated by a single individual, chosen uniformly at random from the population, becoming infected, with the other individuals in the population all assumed to be susceptible. The infectious periods of different infectives are each distributed according to a random variable *I*, having an arbitrary but specified distribution, which is most conveniently specified in terms of its moment generating function $\phi _I (\theta ) = E[\mathrm {e}^{-\theta I} ]$ ($\theta \ge 0$). Throughout its infectious period, a given infective makes infectious contact with any given member of its household at the points of a Poisson process having rate $\lambda _L$ and with any given global neighbour at the points of a Poisson process with rate $\lambda _G$. (Note that $\lambda _L$ and $\lambda _G$ are both individual to individual contact rates.) If an individual so contacted is susceptible then it becomes infected, otherwise the contact has no effect. Contacted susceptibles are immediately able to infect other individuals. An infective individual becomes removed at the end of its infectious period and plays no further role in the epidemic. All infectious periods, global degrees and Poisson processes are assumed to be mutually independent. The epidemic ceases as soon as there is no infective in the population.

For ease of exposition we assume throughout that there is no latent period and that the epidemic is started by a single infective chosen uniformly at random from the population. As explained by Ball et al. (2010), these assumptions may be relaxed without compromising mathematical tractability. In particular, our results are related to the final outcome of the epidemic, the distribution of which is invariant to very general assumptions concerning a latent period (see e.g. Pellis et al. 2008).

### Threshold behaviour

#### Early stages of epidemic

Recall that the global network is formed by pairing up the half-edges uniformly at random. It follows that in the early stages of an epidemic the probability that a global contact is with an individual residing in a previously infected household is small, indeed it is zero in the limit as $m \rightarrow \infty$. Thus, in the early stages of an epidemic, the process of infected households can be approximated by a branching process. The individuals in this branching process correspond to infected households in the epidemic process, and the offspring of a given individual in the branching process are all households that are contacted globally by members of the local (within-household) epidemic in the parent household.

The offspring distribution for this branching process is usually different in the initial generation than in all subsequent generations. The number of global neighbours that the initial infective in the population may infect is distributed according to *D*, as that individual is chosen uniformly at random from the population. The number of global neighbours that the initial infective in any subsequent infected household (in the branching process approximation) may infect is distributed according to ${\tilde{D}}-1$, where ${\tilde{D}}$ is the degree of a typical neighbour of a typical individual in the network. The $-1$ arises because such an infective has been infected through the global network, so one of its neighbours (i.e. its infector) is not available for further infection. Note that a given half-edge is *k* times as likely to be paired with a half-edge emanating from an individual with degree *k* than with one emanating from an individual with degree 1, so $P({\tilde{D}}=k)=\mu _D^{-1} k p_k$ ($k=1,2,\ldots$). The distributions of *D* and ${\tilde{D}}-1$ are equal if and only if *D* has a Poisson distribution. For any non-negative integer valued random variable *X*, we denote its probability generating function (PGF) by $f_X$, so $f_X(s) = E[s^X]$ ($0 \le s \le 1$). We note for future reference that $f_{{\tilde{D}}-1}(s) = f'_D(s)/\mu _D$.

The approximation of the early stages of the epidemic process by the above branching process is made mathematically fully rigorous by Ball et al. (2009) and Ball and Sirl (2012). The latter shows that a sequence of epidemic processes, indexed by *m*, and the approximating branching process can be constructed on the same probability space so that, as $m \rightarrow \infty$, the number of households ultimately infected in the epidemic process converges almost surely to the total progeny of the branching process. Thus, provided *m* is large, whether or not the epidemic can become established and lead to a major outbreak is determined by whether or not the branching process is supercritical.

Let *C* and ${\tilde{C}}$ be random variables describing the number of offspring of the initial and a typical subsequent individual, respectively, in the branching process. Then standard branching process theory (e.g. Haccou et al. 2005, Theorem 5.2), gives that the extinction probability of the branching process is strictly less than one if and only if $R_{*} = E[{\tilde{C}}] > 1$. Thus $R_{*}$ serves as a threshold parameter for the epidemic model. We now outline the calculation of $R_{*}$. Further details are given by Ball et al. (2010).

First note that, since the degree and household size of an individual are independent, the probability that a typical globally infected individual resides in a household of size *n* is given by ${\tilde{\rho }}_n = \mu _H^{-1} n \rho _n$ ($n=1,2,\ldots$). (An individual chosen uniformly at random from the population is *n* times as likely to reside in a given household of size *n* than in a given household of size 1.) Thus,

2.1 $$\begin{aligned} E[{\tilde{C}}]=\sum _{n=1}^{\infty } {\tilde{\rho }}_n E\left[ {\tilde{C}}^{(n)}\right] , \end{aligned}$$

where ${\tilde{C}}^{(n)}$ is the number of global infections emanating from a typical size-*n* single-household epidemic initiated by a single infective who is infected through the global network. Consider such a size-*n* single-household epidemic. Label the household members $0,1,\ldots ,n-1$, where 0 is the initial infective, and write

2.2 $$\begin{aligned} {\tilde{C}}^{(n)}=C_0 + \sum _{i=1}^{n-1} \chi _i C_i , \end{aligned}$$

where $\chi _i=1$ if individual *i* is infected by the single-household epidemic, otherwise $\chi _i=0$, and $C_i$ is the number of global infections made by individual *i* (assuming it is infected). (Throughout the paper we adopt the usual convention that empty sums are equal to 0, so, for example, ${\tilde{C}}^{(1)}=C_0$.) Let $T^{(n)}=\sum _{i=1}^{n-1} \chi _i$ be the final size of the single-household epidemic, not including the initial infective, and $\mu ^{(n)} (\lambda _L)=E[T^{(n)}]$. (A formula for $\mu ^{(n)} (\lambda _L)$ is given in Eq. (B.25) in Appendix B.6.) Whether or not a given individual, *i* say, is infected by the single-household epidemic is independent of its infectious period, so $\chi _i$ and $C_i$ are independent. Thus, taking expectations of (2.2) and exploiting symmetries yields

2.3 $$\begin{aligned} E\left[ {\tilde{C}}^{(n)}\right] = E[C_0] + \mu ^{(n)} (\lambda _L ) E[C_1]. \end{aligned}$$

Since the probability that a Poisson process of rate $\lambda _G$ has no points in a time of length *I* is $\mathrm {e}^{-\lambda _GI}$ we have that the probability an infected individual infects a given global neighbour is $p_G = 1 - E[ \mathrm {e}^{-\lambda _G I}] = 1-\phi _I(\lambda _G)$. We also have that the number of uninfected global neighbours of individual 0 is distributed according to ${\tilde{D}}-1$. Thus $E[C_0]=p_G \mu _{{\tilde{D}}-1}$, where $\mu _{{\tilde{D}}-1}=E[{\tilde{D}}-1]=\mu _D + \frac{\sigma _D^2}{\mu _D} - 1$. Similarly, $E[C_1]=p_G \mu _D$, since the number of uninfected global neighbours of individual 1 is distributed according to *D*. Substituting these results into (2.3) and then into (2.1) yields

2.4 $$\begin{aligned} R_{*} = p_G \left[ \mu _{{\tilde{D}}-1} + \mu _D \sum _{n=1}^{\infty } {\tilde{\rho }}_n \mu ^{(n)} (\lambda _L) \right] . \end{aligned}$$

Let $p_{{\mathrm {maj}}}$ be the probability that a major outbreak occurs. Then standard branching process theory [e.g. Haccou et al. (2005, Theorem 5.2)], shows that $p_{{\mathrm {maj}}}=1-f_C (\sigma )$, where $\sigma$ is the smallest solution of $f_{{\tilde{C}}} (s)=s$ in [0, 1]. Note that, analagous to (2.1), $f_C(s)=\sum _{i=1}^{\infty } {\tilde{\rho }}_n f_{C^{(n)}}(s)$ and $f_{{\tilde{C}}} (s) = \sum _{n=1}^{\infty } {\tilde{\rho }}_n f_{{\tilde{C}}^{(n)}} (s)$. The PGFs $f_{C^{(n)}}$ and $f_{{\tilde{C}}^{(n)}}$ can be calculated using the methods described in Appendices B.1 and B.2. As noted by Ball et al. (2010, Sect. 3.2), these calculations are much simpler in the case where the infectious period is constant, since in this case an infected individual infects its global neighbours independently.

#### Final outcome of major outbreak

We now consider the fraction of the population that are ultimately infected by a major outbreak. The key tool we use is the *susceptibility set* (Ball 2000; Ball and Lyne 2001; Ball and Neal 2002), which we now describe. Let $\mathcal {N}=\{1,2,\ldots ,N\}$ denote the entire population of *N* individuals. For each $i \in \mathcal {N}$, by sampling from the infectious period distribution and then the relevant Poisson processes for local and global contacts, draw up a (random) list of individuals *i* would make infectious contact with if it was to become infected. Then construct a random directed graph on $\mathcal {N}$, in which for any pair, (*i*, *j*) say, of individuals there is an arc from *i* to *j* if and only if *j* is in *i*’s list. The susceptibility set of a given individual, *i* say, consists of those individuals from which there is a chain of arcs to *i* in the graph (including *i* itself). Note that any given individual is ultimately infected by the epidemic if and only if the initial infective belongs to its susceptibility set.

Similarly to the early stages of an epidemic, we can approximate the susceptibility set of an individual, *i* say, chosen uniformly at random from the population, by a households-based branching process. We first consider *i*’s *local* susceptibility set, i.e. the susceptibility set obtained when only local (within-household) contacts are considered. Suppose that this local susceptibility set has size $M^{(n)} +1$, where *n* denotes the size of *i*’s household; so $M^{(n)}$ is the size of the local susceptibility set excluding *i*. (The probability mass function of $M^{(n)}$ is given by Eq. (B.23) in Appendix B.5.) Let *B* be the number of individuals, who are not in *i*’s household, that in the random directed graph have an edge leading directly *to* one of the $M^{(n)} +1$ individuals in *i*’s local susceptibility set. Each individual in *i*’s local susceptibility set has global degree distributed independently according to *D*, and, as $m \rightarrow \infty$, each global neighbour of *i*’s local susceptibility set enters *i*’s susceptibility set independently with probability $p_G$. Thus, taking expectations with respect to *i*’s household size,

2.5 $$\begin{aligned} f_B (s) = \sum _{n=1}^{\infty } {\tilde{\rho }}_n f_{B^{(n)}} (s), \end{aligned}$$

where

2.6 $$\begin{aligned} f_{B^{(n)}} (s) = f_D (1-p_G + p_G s) f_{M^{(n)}} (f_D (1-p_G + p_G s)). \end{aligned}$$

The above *B* individuals form the first generation of our approximating branching process. We next consider each of these *B* individuals in turn, construct the local susceptibility sets in their respective households (which are distinct with probability tending to one as $m \rightarrow \infty$) and then examine the global neighbours of these local susceptibility sets to obtain the second generation of the approximating branching process, and so on. Note that the initial individual in each of these *B* local susceptibility sets has degree distributed according to ${\tilde{D}}$ and one of its global neighbours is already in *i*’s susceptibility set. Thus, as above, the offspring distribution for the branching process is different for the initial individual than for all subsequent individuals. If we let ${\tilde{B}}$ denote the offspring random variable of a typical non-initial individual, then arguing as in the derivation of (2.5),

$$\begin{aligned} f_{{\tilde{B}}} (s) = \sum _{n=1}^{\infty } {\tilde{\rho }}_n f_{{\tilde{B}}^{(n)}} (s), \end{aligned}$$

where

2.7 $$\begin{aligned} f_{{\tilde{B}}^{(n)}} (s) = f_{{\tilde{D}}-1} (1-p_G + p_G s) f_{M^{(n)}} (f_D (1-p_G + p_G s)). \end{aligned}$$

The probability that the approximating branching process survives (i.e. does not go extinct) is given by $z=1-f_B (\xi )$, where $\xi$ is the smallest solution of $f_{{\tilde{B}}} (s)=s$ in [0, 1]. It is straightforward to show that $E[{\tilde{B}}]=E[{\tilde{C}}]$, so $z > 0$ if and only if $R_{*}>1$. Moreover, if $R_{*} > 1$ then *z* is the expected proportion of the population that is ultimately infected by a major outbreak in the limit as $m \rightarrow \infty$; see Ball et al. (2009) for a formal proof when all the households have the same size. Furthermore, the proof of Ball et al. (2014, Theorem 3.4) can be adapted to show that, as $m \rightarrow \infty$, the proportion of the population that is ultimately infected by a major outbreak converges in probability to *z*. Thus we refer to *z* as the relative final size of a major outbreak.

### Vaccination

In modelling vaccination there are two distinct aspects that must be modelled: who gets vaccinated and what happens to those who are vaccinated. Vaccine *allocation* models (addressing the former issue) are our focus in this paper. We now outline the vaccine *action* models (addressing the latter aspect) that we allow for in our analysis.

We use a model for vaccine action, proposed by Becker and Starczak (1998), in which the vaccine response of an individual who is vaccinated is described by a random vector (*A*, *B*) which takes values in $[0,\infty )^2$. Here *A* denotes the relative susceptibility (compared to an unvaccinated individual) and *B* the relative infectivity if the vaccinated individual becomes infected. Thus all Poisson processes concerning potential infection of the individual have their rates multiplied by *A* and the Poisson processes governing the contacts the individual makes, if infected, have their rates multiplied by *B*. The vaccine responses of distinct vaccinees are assumed to be mutually independent. Within this framework we consider two special cases, the *all-or-nothing* and the *non-random* vaccine responses, where (*A*, *B*) is supported on 2 and 1 points respectively. Our methods extend to vaccine action models where (*A*, *B*) is supported on finitely many points; the extension is straightforward analytically but quickly becomes numerically cumbersome if there are more than a few possible outcomes of (*A*, *B*).

The all-or-nothing model (see e.g. Halloran et al. 1992) is obtained by setting $P((A,B)=(0,0))=1-P((A,B)=(1,1))=\varepsilon$, so vaccinated individuals are rendered completely immune with probability $\varepsilon$, otherwise the vaccine has no effect. The non-random model assumes that $P((A,B)=(a,b))=1$, for some (*a*, *b*), so all vaccinated individuals respond identically (see e.g. Ball and Lyne 2006). An important special case is the *leaky* model (see e.g. Halloran et al. 1992), when $b=1$, so vaccination does not affect an individual’s ability to transmit the disease if they become infected. Setting $\varepsilon =1$ in the all-or-nothing model or $a=b=0$ in the non-random model yields a *perfect* vaccine response; that is one in which all vaccinated individuals are rendered completely immune.

## Households based vaccination

### Introduction

In this section we consider vaccine allocation strategies based on household size and determine their impact on $R_{*}$, $p_{{\mathrm {maj}}}$ and *z* under the all-or-nothing and non-random vaccine action models. For $n=1,2,\ldots$ and $v=0,1,\ldots ,n$, let $x_{nv}$ denote the proportion of households of size *n* that have *v* members vaccinated. Let $p_V$ denote the proportion of the population that is vaccinated, i.e. the *vaccination coverage*. Then $p_V$ is also the probability that an individual chosen uniformly at random from the population is vaccinated. Conditioning on the size of such an individual’s household yields

3.1 $$\begin{aligned} p_V = \sum _{n=1}^{\infty } {\tilde{\rho }}_n \sum _{v=0}^n \frac{v}{n} x_{nv}. \end{aligned}$$

We derive results for an arbitrary but specified vaccine allocation however in the numerical studies we consider four allocation schemes: uniformly chosen households, uniformly chosen individuals, ‘best’ and ‘worst’. In the uniformly chosen households scheme, households are chosen uniformly at random and all of their members are vaccinated. Thus, if the vaccination coverage is $p_V$, $x_{nv}=p_V \delta _{vn}+(1-p_V)\delta _{v0}$ ($n=1,2,\ldots$; $v=0,1,\ldots ,n$), where $\delta _{vk}=1$ if $v=k$ and $\delta _{vk}=0$ if $v \ne k$. In the uniformly chosen individuals scheme, individuals are chosen uniformly at random and vaccinated, so $$x_{nv} = \left( {\begin{array}{c}n\\ v\end{array}}\right) p_V^v (1-p_V)^{n-v}$$ ($n=1,2,\ldots$; $v=0,1,\ldots ,n$). The best and worst schemes are the allocations that make $R_{*}$ respectively as small as possible and as large as possible, for a given vaccination coverage.

### All-or-nothing vaccine action

To analyse the consequences of a vaccination scheme using an all-or-nothing vaccine action model it is convenient to use the concept of a potential infectious global contact. Consider a given infected individual, *i* say, and a given global neighbour *j* of *i*. Then *j* is a potential infectious global contact of *i* if it is in *i*’s list of individuals it makes infectious contact with (see the start of Sect. 2.2.2). The potential infectious global contact becomes an actual infectious global contact if *j* is unvaccinated or is vaccinated but the vaccination fails.

The early stages of an epidemic with vaccination are approximated by a branching process of (potentially) infected households, and the offspring of a given individual in the branching process are all households with which members of the single-household epidemic in the parent household make a potential global infectious contact. As in the case without vaccination, the offspring distribution of this branching process is usually different in the initial generation from all subsequent generations. Let ${\tilde{C}}'$ denote the offspring random variable for a non-initial individual. Conditioning first on the size of the corresponding household and then on the number of people vaccinated in that household yields, in an obvious notation,

3.2 $$\begin{aligned} f_{{\tilde{C}}'} (s) = \sum _{n=1}^{\infty } {\tilde{\rho }}_n \sum _{v=0}^n x_{nv} f_{{\tilde{C}}_{n,v}'} (s). \end{aligned}$$

To determine $f_{{\tilde{C}}_{n,v}'} (s)$, consider a household in state (*n*, *v*), i.e. of size *n* having *v* members vaccinated. For $k=0,1,\ldots ,v$, the probability that *k* vaccinations are successful is $$\left( {\begin{array}{c}v\\ k\end{array}}\right) \varepsilon ^k (1-\varepsilon )^{v-k}$$, and given that *k* vaccinations are successful, the probability that the initial potentially contacted individual in that household is susceptible and thus triggers a local epidemic is $\frac{n-k}{n}$. Moreover, if such a local epidemic is triggered, the number of potential infectious global contacts emanating from the local epidemic is distributed as ${\tilde{C}}^{(n-k)}$, where ${\tilde{C}}^{(n)}$ is as in Eq. (2.1). Thus,

3.3 $$\begin{aligned} f_{{\tilde{C}}_{n,v}'} (s) = \sum _{k=0}^{v} \left( {\begin{array}{c}v\\ k\end{array}}\right) \varepsilon ^k (1-\varepsilon )^{v-k} \left[ \frac{(n-k)}{n} f_{{\tilde{C}}^{(n-k)}} (s) + \frac{k}{n} \right] . \end{aligned}$$

The distribution of the offspring random variable, $C'$ say, for the initial generation depends on how the initial infective is chosen. For $n=1,2,\ldots$ and $v=0,1,\ldots ,n$, let $p_{n,v}^V$ be the probability that a vaccinated individual chosen uniformly at random resides in a household in state (*n*, *v*) and let $p_{n,v}^U$ be the corresponding probability for an unvaccinated individual. Then

3.4 $$\begin{aligned} p_{n,v}^V = \frac{{\tilde{\rho }}_n x_{nv} \frac{v}{n}}{p_V} \quad \text {and} \quad p_{n,v}^U = \frac{{\tilde{\rho }}_n x_{nv} \left( 1 - \frac{v}{n} \right) }{1-p_V}. \end{aligned}$$

Thus, if the epidemic is started by an individual chosen uniformly at random from all unvaccinated individuals being infected, then $C'$ is distributed as $C_U '$, where

$$\begin{aligned} f_{C_U '} (s) = \sum _{n=1}^{\infty } \sum _{v=0}^{n-1} p_{n,v}^U \sum _{k=0}^v \left( {\begin{array}{c}v\\ k\end{array}}\right) \varepsilon ^k (1-\varepsilon )^{v-k} f_{C^{(n-k)}} (s). \end{aligned}$$

Alternatively, if the epidemic is started by choosing a vaccinated individual uniformly at random, who triggers an outbreak only if its vaccination fails, then $C'$ is distributed as $C_V '$, where

$$\begin{aligned} f_{C_V '} (s) = \varepsilon + (1- \varepsilon ) \sum _{n=1}^{\infty } \sum _{v=1}^n p_{n,v}^V \sum _{k=0}^{v-1} \left( {\begin{array}{c}v-1\\ k\end{array}}\right) \varepsilon ^k (1-\varepsilon )^{v-1-k} f_{C^{(n-k)}} (s). \end{aligned}$$

The probability of a major outbreak may now be calculated as at the end of Sect. 2.2.1. Again, the formulae simplify appreciably if the infectious period is constant.

A post-vaccination threshold parameter is given by $R_v = E[{\tilde{C}} ']= f_{{\tilde{C}} '} ' (1)$. Differentiating (3.2) and (3.3), or a direct calculation, yields

3.5 $$\begin{aligned} R_v = \sum _{n=1}^{\infty } {\tilde{\rho }}_n \sum _{v=0}^n x_{nv} \mu _{n,v}, \end{aligned}$$

where $\mu _{n,v} = E[ {\tilde{C}}_{n,v} ' ]$ is given by

$$\begin{aligned} \mu _{n,v} = \sum _{k=0}^v \left( {\begin{array}{c}v\\ k\end{array}}\right) \varepsilon ^k (1-\varepsilon )^{v-k} \left( \frac{n-k}{k} \right) E[ {\tilde{C}}^{(n-k)} ]. \end{aligned}$$

As with the case of no vaccination, we can determine the relative final size of a major outbreak by considering a households-based branching process that approximates the susceptibility set of a typical individual. As with the forward process, it is convenient to consider potential global neighbours when constructing this branching process. Thus we start with an individual, $i^*$ say, chosen uniformly at random from the population, construct its local susceptibility set, taking the vaccine status of individuals in the household into account, then determine which global neighbours of individuals in this local susceptibility set would enter the susceptibility set of $i^{*}$ if they were susceptible (i.e. unvaccinated or unsuccessfully vaccinated). These individuals correspond to the first generation of the approximating backward branching process. Suppose that there are $B'$ such individuals. Next we take each of these $B'$ individuals in turn, first determine whether they really do enter the susceptibility set of $i^{*}$ (this happens with probability 1 if the individual is unvaccinated and with probability $1-\varepsilon$ if it is vaccinated, independently for distinct individuals) and if they do enter the susceptibility set of $i^{*}$, determine the number of potential global neighbours of its local susceptibility set to obtain its offspring in the branching process, and so on.

Let ${\tilde{B}} '$ be the offspring random variable for any non-initial individual in this backward branching process. Conditioning first on the state (*n*, *v*) of that individual’s household and then on whether it joins the susceptibility set of $i^{*}$, we obtain

$$\begin{aligned} f_{{\tilde{B}}'} (s) = \sum _{n=1}^{\infty } {\tilde{\rho }}_n \sum _{v=0}^n x_{nv} f_{{\tilde{B}}_{n,v}'} (s), \end{aligned}$$

where

$$\begin{aligned} f_{{\tilde{B}}_{n,v}'} (s) = \sum _{k=0}^v \left( {\begin{array}{c}v\\ k\end{array}}\right) \varepsilon ^k (1-\varepsilon )^{v-k} \left[ \frac{(n-k)}{n} f_{{\tilde{B}}^{(n-k)}} (s) + \frac{k}{n} \right] \end{aligned}$$

and $f_{{\tilde{B}}^{(n)}}$ is as in Eq. (2.7).

The distribution of $B'$ depends on how the initial individual $i^{*}$ is chosen. If $i^{*}$ is chosen uniformly at random from all unvaccinated individuals, then $B'$ is distributed as $B_U '$, say, and conditioning on the state (*n*, *v*) of $i^{*}$’s household yields

$$\begin{aligned} f_{B_U '} (s) = \sum _{n=1}^{\infty } \sum _{v=0}^{n-1} p_{n,v}^U \sum _{k=0}^v \left( {\begin{array}{c}v\\ k\end{array}}\right) \varepsilon ^k (1-\varepsilon )^{v-k} f_{B^{(n-k)}} (s), \end{aligned}$$

where $f_{B^{(n)}}$ is as in Eq. (2.6). If $i^{*}$ is chosen uniformly at random from all vaccinated individuals, then $B'$ is distributed as $B_V '$, where

$$\begin{aligned} f_{B_V '} (s) = \varepsilon + (1- \varepsilon ) \sum _{n=1}^{\infty } \sum _{v=1}^n p_{n,v}^V \sum _{k=0}^{v-1} \left( {\begin{array}{c}v-1\\ k\end{array}}\right) \varepsilon ^k (1-\varepsilon )^{v-1-k} f_{B^{(n-k)}} (s). \end{aligned}$$

Let $\xi ^V$ be the smallest solution of $f_{{\tilde{B}} '} (s)=s$ in [0, 1]. Then the proportion of unvaccinated individuals that are ultimately infected by a major outbreak is $z^U = 1-f_{B_U '} ( \xi ^V )$ and the corresponding proportion for vaccinated individuals is $z^V = 1-f_{B_V '} (\xi ^V )$. The overall proportion of the population infected by a major outbreak is $z=p_V z^V + (1-p_V) z^U$.

### Non-random vaccine action

Analysing the consequences of a vaccination scheme using a non-random vaccine action model is more difficult than with an all-or-nothing vaccine action model since disease spread is now genuinely two type. Thus we now consider two types of individual: type-*U*, unvaccinated, and type-*V*, vaccinated. The early stages of the epidemic can again be approximated by a branching process of infected households. This is now a two-type branching process, with the type of an infected household being given by the type of the initial case in that household. The offspring of a given individual in the branching process correspond to the households that are contacted globally by members of that individual’s corresponding single-household epidemic in the epidemic process.

Let ${\varvec{C}}^U = (C_{UU}, C_{UV})$ denote the offspring random variable for the initial individual in the above branching process, given that individual is of type *U*, and let ${\varvec{C}}^V = (C_{VU}, C_{VV})$ be the corresponding offspring random variable when the initial individual has type *V*. Thus, for example, $C_{UU}$ and $C_{UV}$ are respectively the number of unvaccinated and vaccinated individuals globally infected by the initially infected household, given that the first infective in that household is unvaccinated. Define ${\tilde{{\varvec{C}}}}^U = ( {\tilde{C}}_{UU}, {\tilde{C}}_{UV})$ and ${\tilde{{\varvec{C}}}}^V = ( {\tilde{C}}_{VU}, {\tilde{C}}_{VV})$ similarly for subsequent individuals in the branching process. For ${\varvec{s}} = (s_U , s_V ) \in [0,1]^2$, let $f_{{\varvec{C}}^U} ({\varvec{s}}) = E[s_U^{C_{UU}} s_V^{C_{UV}} ]$ and define $f_{{\varvec{C}}^V} ({\varvec{s}})$, $f_{{\tilde{{\varvec{C}}}}^U} ({\varvec{s}})$ and $f_{{\tilde{{\varvec{C}}}}^V} ({\varvec{s}})$ similarly. (Here and henceforth, for any discrete random vector ${\varvec{X}}$ we denote its joint PGF by $f_{{\varvec{X}}}$.) Recall that when the network of global contacts is formed half-edges are paired uniformly at random. It follows that, for $A \in \{ U,V \}$,

$$\begin{aligned} f_{{\tilde{{\varvec{C}}}}^A} ({\varvec{s}})=\sum _{n=1}^{\infty } \sum _{v=0}^n p_{n,v}^A f_{{\tilde{{\varvec{C}}}}_{n,v}^A} ({\varvec{s}}), \end{aligned}$$

where ${\tilde{{\varvec{C}}}}_{n,v}^A$ is a random vector giving the numbers of unvaccinated and vaccinated global infections that emanate from a non-initial infected household of size *n*, having *v* members vaccinated, whose primary case is of type *A*. The distributions of ${\varvec{C}}^U$ and ${\varvec{C}}^V$ depend on how the initial infective is chosen. If it is chosen uniformly at random from all individuals of the appropriate type in the population, then, for $A \in \{ U,V \}$,

$$\begin{aligned} f_{{\varvec{C}}^A} ({\varvec{s}}) = \sum _{n=1}^{\infty } \sum _{v=0}^n p_{n,v}^A f_{{\varvec{C}}_{n,v}^A} ({\varvec{s}}), \end{aligned}$$

where ${\varvec{C}}_{n,v}^A$ is defined analogously to ${\tilde{{\varvec{C}}}}_{n,v}^A$ but for the initial infected household.

Let

$$\begin{aligned} {\tilde{M}} = \left[ \begin{array}{ll}{\tilde{m}}_{UU}&{}{\tilde{m}}_{UV}\\ {\tilde{m}}_{VU}&{}{\tilde{m}}_{VV}\end{array}\right] , \end{aligned}$$

where, for example, $${\tilde{m}}_{UU} = E[ {\tilde{C}}_{UU} ]$$. The post-vaccination threshold parameter $$R_v$$ is given by the dominant eigenvalue (a real, positive eigenvalue of maximum modulus) of $${\tilde{M}}$$. It is well known (e.g. Haccou et al. 2005, p. 123) that, provided $${\tilde{m}}_{UV} {\tilde{m}}_{VU} \ne 0$$, the two-type branching process has non-zero probability of surviving if and only if $$R_v > 1$$. Moreover, if $${\tilde{m}}_{UV} {\tilde{m}}_{VU} \ne 0$$ and $$R_v > 1$$, then the survival probability (and hence the probability of a major outbreak) can be determined as follows. Let $${\varvec{\sigma }} = (\sigma _U, \sigma _V )$$ be the unique solution in $$[0,1)^2$$ of the equations

3.6 $$\begin{aligned} \sigma _U = f_{{\tilde{C}}^U} (\sigma _U, \sigma _V), \quad \sigma _V = f_{{\tilde{C}}^V} (\sigma _U, \sigma _V). \end{aligned}$$

Then, if the epidemic is started by an unvaccinated individual chosen uniformly at random from the population becoming infected, the probability of a major outbreak is $p_{{\mathrm {maj}}}^U = 1-f_{C^U} ({\varvec{\sigma }})$. The corresponding probability when the initial infective is a vaccinated individual is $p_{{\mathrm {maj}}}^V = 1 - f_{C^V} ({\varvec{\sigma }})$. Calculation of the PGFs $f_{{\varvec{C}}^U_{n,v}}$, $f_{{\varvec{C}}^V_{n,v}}$, $f_{{\tilde{{\varvec{C}}}}^U_{n,v}}$ and $f_{{\tilde{{\varvec{C}}}}^V_{n,v}}$, which is rather involved unless the infectious period is constant, requires the methods described in Appendices B.1 and B.3. Calculation of ${\tilde{M}}$, which is sufficient for determining optimal vaccination strategies, is simpler and we now outline it.

Consider first a local (single-household) epidemic in a household in state (*n*, *v*), initiated by one of the household members becoming infected. Let $T_U^{(n,v)}$ and $T_V^{(n,v)}$ denote the number of unvaccinated and vaccinated individuals ultimately infected by this local epidemic, not including the initial case. For $A,A' \in \{ U,V \}$, let $\mu ^{(n,v)} (A,A')$ be the mean of $T_{A'}^{(n,v)}$ given that the initial case is of type *A*. (Calculation of $\mu ^{(n,v)} (A,A')$ is described in Appendix B.6.) Also, define the vaccine-status-dependent marginal transmission probabilities $p_G^{\mathrm{NR}} (U,U)$, $p_G^{\mathrm{NR}} (U,V)$, $p_G^{\mathrm{NR}} (V,U)$ and $p_G^{\mathrm{NR}} (V,V)$ between global neighbours, where, for example, $p_G^{\mathrm{NR}} (U,V)$ is the probability that an unvaccinated infective infects a given vaccinated global neighbour. Then

3.7 $$\begin{aligned} P_G^{\mathrm{NR}} = \left[ \begin{array}{ll} p_G^{\mathrm{NR}} (U,U)&{}\quad p_G^{\mathrm{NR}} (U,V)\\ p_G^{\mathrm{NR}} (U,V)&{}\quad p_G^{\mathrm{NR}} (V,V)\end{array}\right] = \left[ \begin{array}{ll}1-\phi _I (\lambda _G)&{}\quad 1-\phi _I (a \lambda _G)\\ 1-\phi _I (b \lambda _G)&{}\quad 1-\phi _I (ab \lambda _G )\end{array}\right] , \end{aligned}$$

recalling that $\phi _I$ is the moment generating function of the generic infectious period random variable *I*.

Conditioning on the state (*n*, *v*) of the infected household shows that, for $A,A' \in \{ U,V\}$,

3.8 $$\begin{aligned} {\tilde{m}}_{AA'} = \sum _{n=1}^{\infty } \sum _{v=0}^n p_{n,v}^A {\tilde{m}}_{AA'}^{(n,v)}, \end{aligned}$$

where ${\tilde{m}}_{AA'}^{(n,v)}$ is defined analogously to ${\tilde{m}}_{AA'}$ but for a household in state (*n*, *v*). Further, arguing as in the derivations of (2.3) and (2.4), yields

3.9 $$\begin{aligned} {\tilde{m}}_{AA'}^{(nv)} = \left[ \mu _{{\tilde{D}}-1} p_G^{\mathrm{NR}} (A,A') + \mu _D \left( \mu ^{(n,v)} (A,U) p_G^{\mathrm{NR}} (U,A') + \mu ^{(n,v)} (A,V) p_G^{\mathrm{NR}} (V,A')\right) \right] p_{A'},\nonumber \\ \end{aligned}$$

where $p_U = 1-p_V$. Hence, if we let

$$\begin{aligned} F=\left[ \begin{array}{ll}F_{UU}&{}F_{UV}\\ F_{VU}&{}F_{VV}\end{array}\right] \quad \text {and}\quad D_V= \left[ \begin{array}{cc}1-p_V&{}0\\ 0&{}p_V\end{array}\right] , \end{aligned}$$

where, for $$A,A' \in \{U,V\}$$,

$$\begin{aligned} F_{AA'}=\sum _{n=1}^{\infty } \sum _{v=0}^n p_{n,v}^A \mu ^{(n,v)}(A,A'), \end{aligned}$$

then (3.8) and (3.9) yield

3.10 $$\begin{aligned} {\tilde{M}}=(\mu _{{\tilde{D}} -1} I + \mu _D F) P_G^{\mathrm{NR}} D_V . \end{aligned}$$

Turning to the relative final size of a major outbreak, we consider a households-based branching process that approximates the susceptibility set of a given individual. This is now a two-type branching process, with type (*U* or *V*) corresponding to the type of the primary member of the corresponding local susceptibility set. Define the offspring random variables ${\varvec{B}}^U = (B_{UU}, B_{UV})$, ${\varvec{B}}^V = (B_{VU}, B_{VV})$, ${\tilde{{\varvec{B}}}}^U = ({\tilde{B}}_{UU}, {\tilde{B}}_{UV})$ and ${\tilde{{\varvec{B}}}}^V = ( {\tilde{B}}_{VU}, {\tilde{B}}_{VV})$ for this branching process in the obvious fashion (cf. the forward process offspring random variables ${\varvec{C}}^U$, ${\varvec{C}}^V$, ${\tilde{{\varvec{C}}}}^U$ and ${\tilde{{\varvec{C}}}}^V$ and the notation used in Sect. 3.2). We determine first the PGFs $f_{{\tilde{{\varvec{B}}}}^U} ({\varvec{s}})$ and $f_{{\tilde{{\varvec{B}}}}^V} ({\varvec{s}})$.

First note that, for $A \in \{ U,V \}$, conditioning on the state (*n*, *v*) of a household yields, in obvious notation,

$$\begin{aligned} f_{{\tilde{{\varvec{B}}}}^A} ({\varvec{s}}) = \sum _{n=1}^\infty \sum _{v=0}^n p_{n,v}^A f_{{\tilde{{\varvec{B}}}}_{n,v}^A} ({\varvec{s}}). \end{aligned}$$

Fix $A \in \{ U,V \}$ and (*n*, *v*), and let ${\varvec{M}}_A^{(n,v)} = (M_{AU}^{(n,v)}, M_{AV}^{(n,v)})$, where $M_{AU}^{(n,v)}$ and $M_{AV}^{(n,v)}$ are respectively the numbers of unvaccinated and vaccinated individuals, not including $i^{*}$ itself, in the local susceptibility set of a typical type-*A* individual, $i^{*}$ say, that resides in a household in state (*n*, *v*). Then ${\tilde{{\varvec{B}}}}_{n,v}^A$ admits the decomposition

3.11 $$\begin{aligned} {\tilde{{\varvec{B}}}}_{n,v}^A = {\tilde{{\varvec{B}}}}_{n,v}^{A0} + \sum _{i=1}^{M_{AU}^{(n,v)}} {\tilde{{\varvec{B}}}}_{n,v}^{AU} (i) + \sum _{j=1}^{M_{AV}^{(n,v)}} {\tilde{{\varvec{B}}}}_{n,v}^{AV} (j), \end{aligned}$$

where ${\tilde{{\varvec{B}}}}_{n,v}^{A0}$, ${\tilde{{\varvec{B}}}}_{n,v}^{AU} (i)$ and ${\tilde{{\varvec{B}}}}_{n,v}^{AV} (j)$ are the contributions to ${\tilde{{\varvec{B}}}}_{n,v}^A$ from the primary individual $i^{*}$, the *i*th secondary unvaccinated member and the *j*th secondary vaccinated member of $i^{*}$’s local susceptibility set, respectively. Let $D_{i^*}$ denote the number of neighbours $i^{*}$ has in the global network, so $D_{i^*} \sim {\tilde{D}}$, and recall that one of these global neighbours is used when $i^*$ joins the susceptibility set process. Thus,

$$\begin{aligned} {\tilde{{\varvec{B}}}}_{n,v}^{A0} = \sum _{k=1}^{D_{i^*}-1} {\varvec{\chi }}_k^A, \end{aligned}$$

where ${\varvec{\chi }}_k^A = (1,0)$ if the *k*th global neighbour of $i^*$ is unvaccinated and joins the susceptibility set process, ${\varvec{\chi }}_k^A = (0,1)$ if this neighbour is vaccinated and joins the susceptibility set process, and ${\varvec{\chi }}_k^A = (0,0)$ otherwise. Note that, independently, each such global neighbour is vaccinated with probability $p_V$, and it joins the susceptibility set process with probability $p_G^{\mathrm{NR}} (U,A)$ if it is unvaccinated and with probability $p_G^{\mathrm{NR}} (V,A)$ if it is vaccinated. Thus

$$\begin{aligned} f_{{\tilde{{\varvec{B}}}}_{n,v}^{A0}} ({\varvec{s}}) = f_{{\tilde{D}}-1} (p^A ({\varvec{s}})), \end{aligned}$$

where

$$\begin{aligned} p^A ({\varvec{s}}) = f_{{\varvec{\chi }}_1^A} ({\varvec{s}}) = 1- (1-p_V) p_G^{\mathrm{NR}} (U,A) (1-s_U) - p_V p_G^{\mathrm{NR}} (V,A) (1-s_V). \end{aligned}$$

A similar argument shows that $f_{{\tilde{{\varvec{B}}}}_{n,v}^{AU} (i)} ({\varvec{s}})=f_D (p^U ({\varvec{s}}))$ and $f_{{\tilde{{\varvec{B}}}}_{n,v}^{AV} (j)} ({\varvec{s}}) =f_D (p^V ({\varvec{s}}))$.

Exploiting the mutual independence of all the random quantities in (3.11) except the components of ${\varvec{M}}_A^{(n,v)}$ then yields

$$\begin{aligned} f_{{\tilde{{\varvec{B}}}}_{n,v}^A} ({\varvec{s}}) = f_{{\tilde{D}}-1} (p^A ({\varvec{s}})) f_{{\varvec{M}}_A^{(n,v)}} (f_D (p^U ({\varvec{s}})), f_D(p^V ({\varvec{s}}))). \end{aligned}$$

Calculation of the mass function of ${\varvec{M}}_A^{(n,v)}$ is described in Appendix B.5.

The distributions of ${\varvec{B}}^U$ and ${\varvec{B}}^V$ depend on how the initial individual for the susceptibility set process is chosen. For $A \in \{ U,V \}$, if this initial individual is chosen uniformly at random from all type-*A* individuals in the population, then

$$\begin{aligned} f_{{\varvec{B}}^A} ({\varvec{s}}) = \sum _{n=1}^\infty \sum _{v=0}^n p_{n,v}^A f_{{\varvec{B}}_{n,v}^A} ({\varvec{s}}), \end{aligned}$$

where

$$\begin{aligned} f_{{\varvec{B}}_{n,v}^A} ({\varvec{s}}) = f_D (p^A ({\varvec{s}})) f_{{\varvec{M}}_A^{(n,v)}} (f_D (p^U ({\varvec{s}})), f_D (p^V ({\varvec{s}}))). \end{aligned}$$

Suppose that $${\tilde{m}}_{UV} {\tilde{m}}_{VU} \ne 0$$ and $$R_v > 1$$. Let $${\varvec{\xi }} = ( \xi _U, \xi _V )$$ be the unique solution in $$[0,1)^2$$ of the equations

$$\begin{aligned} \xi _U = f_{{\tilde{{\varvec{B}}}}^U} ( \xi _U, \xi _V ), \quad \xi _V = f_{{\tilde{{\varvec{B}}}}^V} ( \xi _U, \xi _V ). \end{aligned}$$

Then the proportions of unvaccinated and vaccinated individuals that are infected by a major epidemic are given by $z^U = 1-f_{{\varvec{B}}^U} (\xi _U, \xi _V)$ and $z^V = 1-f_{{\varvec{B}}^V} (\xi _U, \xi _V)$, respectively. Thus the overall proportion of individuals infected by a major epidemic is $z=p_V z^V + (1-p_V)z^U$.

### Optimal vaccination strategies

A main aim of a vaccination scheme is to reduce the threshold parameter $R_{*}$ to below one, i.e. to make $R_v \le 1$, and thus prevent a major outbreak occurring. The vaccine response may be such that $R_v > 1$ even if the entire population is vaccinated, in which case vaccination by itself is insufficient to be sure of preventing a major outbreak. However, if $R_{*} > 1$ and it is possible to make $R_v \le 1$ then it is of interest to determine the allocation of vaccines that reduces $R_v$ to 1 with the minimum vaccination coverage $p_V$.

Suppose that the population has a maximum household size $n_{\max } < \infty$. Then $p_V$ is a linear function of $x_{nv}$ ($n=1,2,\ldots$, $n_{\max }$; $v=0,1,\ldots ,n$) (recall (3.1)), as is $R_v$ when the vaccine action is all-or-nothing (recall (3.5)). Thus in this case determining the allocation of vaccines that (i) minimises $p_V$ subject to $R_v \le 1$ or (ii) minimises $R_v$ subject to an upper bound on $p_V$ are both linear programming problems. Moreover, as we outline below, the method of Ball and Lyne (2002, 2006) can be used to construct explicitly the solutions of these linear programming problems. The situation is in general more complicated if the vaccine action is non-random, since then $R_v$ is the dominant eigenvalue of the matrix ${\tilde{M}}$, and the corresponding optimisation problems are non-linear. However, the problem is linear if $\text {rank} ({\tilde{M}})=1$, a sufficient condition for which is $\text {rank} (P_G^{\mathrm{NR}})=1$, i.e. $p_G^{\mathrm{NR}} (U,U) p_G^{\mathrm{NR}} (V,V) = p_G^{\mathrm{NR}} (U,V) p_G^{\mathrm{NR}} (V,U)$. Note that $\text {rank} (P_G^{\mathrm{NR}})=1$ if either $a=1$ or $b=1$, so a leaky vaccine response satisfies this constraint.

Consider the non-random vaccine response and suppose that $\text {rank} ({\tilde{M}})=1$. Then $R_v = \text {trace} ({\tilde{M}})$ and, recalling (3.10), it follows using (3.4), (3.8) and (3.9) that

3.12 $$\begin{aligned} R_v = \sum _{n=1}^\infty \sum _{v=0}^n {\tilde{\rho }}_n x_{nv} \mu _{n,v}^{\mathrm{NR}}, \end{aligned}$$

where

$$\begin{aligned} \mu _{n,v}^{\mathrm{NR}}&= \left( 1 - \frac{v}{n} \right) \left\{ {{\mu }}_{\tilde{D}-1} p_G^{\mathrm{NR}} (U,U) + \mu _D \left[ \mu ^{(n,v)} (U,U) p_G^{\mathrm{NR}} (U,U)+ \,\mu ^{(n,v)}(U,V) p_G^{\mathrm{NR}} (V,U)\right] \right\} \nonumber \\&\quad + \frac{v}{n} \left\{ {{\mu }}_{\tilde{D}-1} p_G^{\mathrm{NR}} (V,V) + \mu _D \left[ \mu ^{(n,v)} (V,U) p_G^{\mathrm{NR}} (U,V) + \,\mu ^{(n,v)} (V,V) p_G^{\mathrm{NR}} (V,V)\right] \right\} . \end{aligned}$$

Observe that, when $\text {rank} ({\tilde{M}})=1$, $R_v$ takes the same form as for the all-or-nothing vaccine response; compare (3.12) and (3.5).

To characterise the optimal vaccination schemes in these cases, it is convenient to consider a finite population of *m* households, with maximum household size $n_{\max }$. Let $m_n = m \rho _n$ be the number of households of size *n* and let $h_{nv} = m_n x_{nv}$ be the number of households in state (*n*, *v*). Then ${\tilde{\rho }}_n = nm_n / N$, where *N* is the total population size, and, writing $\mu _{n,v}$ for $\mu _{n,v}^\mathrm{AoN}$ or $\mu _{n,v}^{\mathrm{NR}}$, as appropriate, (3.5) or (3.12) implies that

3.13 $$\begin{aligned} R_v = \sum _{n=1}^{n_{\max }} \sum _{v=0}^n h_{nv} M_{n,v}, \end{aligned}$$

where $M_{n,v}=n \mu _{n,v} / N$, and (3.1) yields

$$\begin{aligned} p_V = \frac{1}{N} \sum _{n=1}^{n_{\max }} \sum _{v=0}^n vh_{nv}. \end{aligned}$$

Observe that (3.13) implies that $R_v$ is obtained by summing $M_{n,v}$ over all households in the population. For $n=1,2,\ldots ,n_{\max }$ and $v=0,1,\ldots ,n-1$, let $G_{n,v}=M_{n,v} - M_{n,v+1}$ be the reduction in $R_v$ obtained by vaccinating one further individual in a household in state (*n*, *v*). If $G_{n,v}$ is decreasing in *v* for each fixed *n* (so successive vaccinations in the same household have diminishing returns), then it is straightforward to determine optimal vaccination schemes (Ball and Lyne 2002, 2006). One simply orders the states (*n*, *v*) according to decreasing $G_{n,v}$, and then uses this ordering to determine the order in which individuals in the population are vaccinated, stopping the process when either the vaccination coverage reaches the desired level or when $R_v \le 1$, depending on the optimisation problem under consideration. (The ‘worst’ scheme is obtained by vaccinating whole households in increasing order of $M_{n,0}-M_{n,n}$.) If for some *n*, say $n=n'$, $G_{n',v}$ is not decreasing in *v*, then only those states, $(n',v')$ say, on the lower edge of the convex hull of $\{ (v,G_{n',v}) :v=0,1,\ldots ,n'-1\}$ can be part of an optimal vaccination scheme. It is still possible to give explicit solutions of associated optimisation problems (cf. Ball et al. 2004), and of ‘worst’ schemes, but the details are more involved.

## Acquaintance vaccination

### Introduction

The acquaintance vaccine allocation model proposed by Cohen et al. (2003) and further analysed by Britton et al. (2007), both in the setting of a standard network model (i.e. a population modelled by a configuration model, without household structure), is as follows. Each individual in the population is sampled independently a Poisson distributed number of times, with mean $\kappa >0$, and each time an individual is sampled it chooses one of its neighbours uniformly at random, with replacement, and that neighbour is vaccinated. If a sampled individual has no neighbours then that sampling is ignored. Individuals are vaccinated at most once, even if they are chosen to be vaccinated more than once.

If the vaccine is perfect then the early stages and final outcome of an epidemic with this acquaintance vaccination model can be analysed relatively easily using branching process approximations. Essentially this is because the epidemic involves only unvaccinated individuals, and the degrees of the neighbours of an unvaccinated individual are mutually independent. However, if the vaccine is imperfect, then the epidemic may also involve vaccinated individuals and the degrees of the neighbours of a vaccinated individual are dependent. (A low-degree neighbour of a given individual, *A* say, is more likely to nominate *A* for vaccination than a high-degree neighbour but, if *A* is vaccinated, at least one of *A*’s neighbours nominated *A* for vaccination.) It follows that the independence property required for a branching process approximation breaks down. This dependence can be overcome if individuals are also typed by their degree but, unless the support of *D* is small, the calculations become computationally prohibitively expensive. Indeed, infinite-type branching processes are required if the support of *D* is countably infinite. For these reasons, Ball and Sirl (2013) introduced an alternative acquaintance vaccine allocation model and analysed it, in the setting of a standard network model (without households). We now extend this analysis to the present network and households model.

### Model and results

#### Acquaintance vaccination model

We assume that each individual is sampled independently with probability $p_S$ and then each sampled individual nominates each of its global neighbours independently with probability $p_N$. All individuals that are nominated at least once are then vaccinated. Thus individuals are sampled only once and it is easily seen that the degrees of the neighbours of both vaccinated and unvaccinated individuals are mutually independent, thus facilitating branching process approximations which do not involve typing by degree. Note that the probability that an individual is not named by a given neighbour is $1-p_S p_N$, so the probability it is vaccinated is

4.1 $$\begin{aligned} p_V = 1 - \sum _{d=0}^{\infty } p_d (1-p_S p_N)^d = 1-f_D (1-p_S p_N ), \end{aligned}$$

which, of course, also gives the vaccination coverage.

We now describe the methods used to find threshold parameter, the major outbreak probability and the relative final size of a major outbreak. All details of the calculations are deferred to Appendix A.

#### Early stages of the epidemic

We approximate the early stages of the epidemic, with vaccination, by a multitype (forward) branching process of infected households, in which households are typed by the type of their primary (globally contacted) case. Such primary cases are typed by (i) whether they are named (*N*), vaccinated (*V*) or unvaccinated (*U*) and (ii) whether or not they are sampled and thus might name their neighbours for vaccination (*S* and $S^c$). Here *N* means that a primary case was named by its global infector and therefore is vaccinated, *V* means that it is not named by its global infector but it is nevertheless vaccinated (i.e. it is named by another neighbour) and *U* means that it is unvaccinated (i.e. not named by any of its neighbours). Thus there are 6 types of infected households, which for notational convenience we give numerical indices as shown in Table 1. Secondary infected cases in a household, and also the primary case in the initially infected household, need only to be typed *V* or *U*, according to whether or not they are vaccinated. This forward branching process is in the same spirit as that described in Sect. 3.3 for household-based vaccination with a non-random vaccine action model, except to maintain the independence required for a branching process there are now 6 rather than 2 types (and an additional 2 types that appear only in the initial generation).

**Table 1:** Numerical indices of the 6 types of household involved in non-initial generations of the branching process approximations

1	2	3	4	5	6
(*N*, *S*)	(*V*, *S*)	(*U*, *S*)	$(N,S^c)$	$(V,S^c)$	$(U,S^c)$

The offspring random variables for this forward branching process are as follows. For $i=1,2,\ldots ,6$, let ${\tilde{{\varvec{C}}}}_i = ({\tilde{C}}_{i1}, {\tilde{C}}_{i2}, \ldots , {\tilde{C}}_{i6})$ denote the offspring random variable for a type-*i* non-initial individual in the forward branching process. Thus ${\tilde{C}}_{ij}$ is the number of type-*j* primary household cases emanating from a typical single household epidemic that is initiated by a single type-*i* primary case. Similarly, for $A\in \{U,V\}$, let ${\varvec{C}}_A = (C_{A1}, C_{A2}, \ldots , C_{A6})$ denote the offspring random variable for a type-*A* individual in the initial generation of the forward branching process. In contrast to similar analyses of previous models in this paper, when analysing the all-or-nothing vaccine response, we consider actual, rather than potential, global infections. This facilitates simultaneous analysis of the two vaccine action models to a much larger extent than is otherwise possible, but has the consequence that the formulae for $p_{{\mathrm {maj}}}$ and *z* below are slightly different for the different models of vaccine action.

A threshold parameter is then given by $R_v$, the dominant eigenvalue of the matrix ${\tilde{M}}=[{\tilde{m}}_{ij}]$, where ${\tilde{m}}_{ij}=E[{\tilde{C}}_{ij}]$. We assume that ${\tilde{M}}$ is positively regular, i.e. that for some $n>0$ the matrix ${\tilde{M}}^n$ has all of its entries strictly positive (e.g. Mode 1971, Sect. 1.6), which will be satisfied in all practical situations. If $R_v>1$ then a major outbreak occurs with strictly positive probability; otherwise the probability of a major outbreak is zero.

The probability of a major outbreak can be written in terms of the joint PGFs $$f_{{\tilde{{\varvec{C}}}}_i}$$ and $$f_{{\varvec{C}}_A}$$ of the random variables $${\tilde{{\varvec{C}}}}_i$$ ($$i=1,2,\ldots ,6$$) and $${\varvec{C}}_A$$ ($$A\in \{U,V\}$$). Let $$p_{{\mathrm {maj}}}^U$$ be the probability of a major outbreak if the initial infective is unvaccinated and $$p_{{\mathrm {maj}}}^V$$ be the corresponding probability when the initial infective is vaccinated. With an all-or-nothing vaccine this latter probability also assumes that the vaccination of the initial case is unsuccessful, since the branching process considers actual global infections. Conditioning on a single initial infective of type $$A\in \{U,V\}$$, we have $$p_{{\mathrm {maj}}}^A=1-f_{{\varvec{C}}_A}({\varvec{\sigma }})$$, where $${\varvec{\sigma }}=(\sigma _i,\,i=1,2,\dots ,6)$$ is the unique solution of $$f_{{\tilde{{\varvec{C}}}}_i}({\varvec{\sigma }})=\sigma _i$$ ($$i=1,2,\dots ,6$$) in $$[0,1)^6$$ (cf. (3.6)). The probability of a major outbreak with an initial infective chosen uniformly from the whole population is therefore

$$\begin{aligned} p_{{\mathrm {maj}}}= {\left\{ \begin{array}{ll} p_V p_{{\mathrm {maj}}}^V + (1-p_V)p_{{\mathrm {maj}}}^U, &{} \quad \text{ non-random } \text{ vaccine, }\\ p_V (1-\varepsilon ) p_{{\mathrm {maj}}}^V + (1-p_V)p_{{\mathrm {maj}}}^U, &{} \quad \text{ all-or-nothing } \text{ vaccine. } \end{array}\right. } \end{aligned}$$

(With an all-or-nothing vaccine, if the initial infective is vaccinated then a major outbreak can occur only if that vaccination is unsuccessful, which occurs with probability $1-\varepsilon$.)

#### Final outcome of a major outbreak

We approximate the susceptibility set of a given individual, $i^{*}$ say, by a households-based (backward) multitype branching process, where, as in Sect. 3.3, the type of a household is given by the type of the primary member of the corresponding local susceptibility set. As previously, the fraction of individuals that are ultimately infected by a major outbreak is given by the survival probability of this branching process. In the initial generation there are two types, *V* and *U*, depending on whether or not $i^{*}$ is vaccinated. In subsequent generations, there are 6 types, numbered 1–6 as above, where now, for example, *N* means that the primary case was named by the individual that it contacts globally to join $i^{*}$’s susceptibility set. Similar to before, secondary members of a local susceptibility set need only to be typed *V* or *U*. Let ${\varvec{B}}_U = (B_{U1}, B_{U2},\ldots ,B_{U6})$ and ${\varvec{B}}_V=(B_{V1},B_{V2},\ldots ,B_{V6})$ denote the offspring random variables for the initial generation of this branching process, and let ${\tilde{{\varvec{B}}}}_i = ({\tilde{B}}_{i1}, {\tilde{B}}_{i2}, \ldots , {\tilde{B}}_{i6})$ ($i=1,2,\ldots ,6$) be the offspring random variables for all subsequent generations.

Suppose that $$R_v > 1$$, so a major outbreak is possible. The asymptotic proportions of unvaccinated and vaccinated individuals that are infected by a major outbreak are given by $$z^U = 1-f_{{\varvec{B}}_U} ({\tilde{{\varvec{\xi }}}})$$ and $$z^V = 1 - f_{{\varvec{B}}_V} ({\tilde{{\varvec{\xi }}}})$$, respectively, where $${\tilde{{\varvec{\xi }}}} = ( {\tilde{\xi }}_1, {\tilde{\xi }}_2, \ldots , {\tilde{\xi }}_6 )$$ is the unique solution in $$[0,1)^6$$ of the equations $$f_{{\tilde{{\varvec{B}}}}_i} ({\varvec{s}}) = s_i$$ ($$i=1,2,\ldots ,6$$). The overall proportion of the population infected by a major outbreak is therefore

$$\begin{aligned} z = {\left\{ \begin{array}{ll} p_V z^V + (1-p_V)z^U, &{} \quad \text{ non-random } \text{ vaccine, }\\ p_V (1-\varepsilon ) z^V + (1-p_V)z^U, &{} \quad \text{ all-or-nothing } \text{ vaccine. } \end{array}\right. } \end{aligned}$$

## Model exploration

In this section we explore the behaviour of the model and its dependence on some key parameters. The main focus is on numerical comparison of the various vaccine allocation regimes for a variety of household size and network degree distributions. First, however, we comment briefly on the relationship between outcomes in simulated finite populations and our asymptotic analytical results, and also give some discussion of the choice of the parameters $p_S$ and $p_N$ in acquaintance vaccination.

### Outcomes in finite populations

One can compare the analytical, asymptotic, quantities of interest ($p_{{\mathrm {maj}}}$ and *z*) to simulation-based estimates of the corresponding quantities on finite populations. We find [consistent with Ball et al. (2009, Sect. 5)] that the agreement becomes reasonable with the number of households *m* in the low hundreds and very close indeed for *m* over about 1000, though the convergence is a little slower for heavier tailed degree distributions.

### Acquaintance vaccination dependence on $p_S$ and $p_N$

The vaccine coverage $c=p_V=1-f_D(1-p_Sp_N)$ in our acquaintance vaccination model is determined by the product $p_Sp_N$ [see Eq. (4.1)]. Here we investigate the dependence of the model outcomes ($R_v$, $p_{{\mathrm {maj}}}$ and *z*) on the precise values of $p_N$ and $p_S$, for a fixed coverage.

In the case of a perfect vaccine some analytical progress is possible. It can be shown (see Appendix A.2.3 for details) that in this case $R_v$ has the form

$$\begin{aligned} R_v = \eta (p') (1-2p'+p'p_N), \end{aligned}$$

where the function $\eta$ depends on $p_S$ and $p_N$ only through their product $p'=p_Sp_N$. It is immediate that $R_v$ is increasing in $p_N$ for fixed $p'$. Thus we have that the best effect is achieved by taking $(p_S,p_N)=(1,p')$ and the worst with $(p_S,p_N)=(p',1)$. The corresponding values of $R_v$ are $R_v^b=\eta (p')(1-p')^2$ and $R_v^w=\eta (p')(1-p')$, so we have $R_v^b = (1-p')R_v^w$. Precisely as in the network-only model without households, we note that this difference is generally quite small and is at its largest when $p'$ is large, so there is high coverage and the epidemic is likely to be subcritical in any event.

When the vaccine action is not perfect we cannot make analytical progress in this direction, but extensive numerical calculations suggest that $R_v$, $p_{{\mathrm {maj}}}$ and *z* are usually monotonic in $p_N$ when $p_Sp_N$ is fixed and that in any case the difference in outcomes between the best and worst choices are very small. We do not explicitly demonstrate this here, but we note that (as might be expected) this is the same as Ball and Sirl (2013) found for the model without households. It can be seen to some extent in all of the plots in Sect. 5.3, where the two acquaintance vaccination plots are barely distinguishable in most cases.

In the numerical studies below we use the terms ‘best’ and ‘worst’ to refer to the acquaintance vaccination schemes with $(p_S,p_N)=(1,p')$ and $(p_S,p_N)=(p',1)$ even though the names are not necessarily correct. As noted above, the precise choice of $p_S$ and $p_N$ seems to have only a very weak influence on the final outcome of the epidemic model and these two cases seem to give bounds for the quantities of interest.

### Effect of different vaccine allocation regimes

We now explore the relative effectiveness of the different vaccine allocation regimes discussed in this paper, across a small but representative variety of household size and degree distributions. In the following figures we plot an outcome ($R_v$ or *z*) against the vaccine coverage *c* for several vaccine allocation strategies; with all other parameters held fixed. Using different plots within the same figure we vary some of these other parameters. The vaccine allocation methods we consider are (i) uniformly chosen individuals (Ind UAR), (ii) uniformly chosen households (HH UAR), (iii & iv) best and worst household-based allocation (HH Best and HH Worst), (v & vi) ‘best’ and ‘worst’ acquaintance vaccination (Acq Best and Acq Worst); see the end of Sect. 3.1 for descriptions of the household-based vaccination schemes.

In this numerical section we use household size distributions $\rho _{{\mathrm {UK}}}=(31,32,16,14,5,2)/100$ and $\rho _{{\mathrm {Pak}}}=(9,45,77,118,141,146,123,104,237)/1000$, which have respective means 2.4 and 6.8, as realistic household size distributions from the UK (Office for National Statistics 2011, Table 3.1) and Pakistan (National Institute of Population Studies 2013, Table 2.9), respectively. The degree distributions we use are the standard Poisson distribution and a power law distribution with exponential cutoff (see, for example, Amaral et al. 2000). For this latter distribution we use the notation $D\sim \text{ PowC }(a,\kappa )$ to denote that $p_k \propto k^{-a} \mathrm {e}^{-k/\kappa }$ ($k=1,2,\dots$), i.e. a power law with index *a* and exponential cutoff at about $\kappa$. In particular, we use the degree distribution $D\sim \text{ PowC }(2,120)$, which has mean $\mu _D\approx 3.001$ and $\sigma ^2_D\approx 66$. We also use the notation $\text{ Gam }(k,r)$ to denote a Gamma distribution with shape parameter *k* and scale parameter *r* (and thus mean *kr* and variance $kr^2$).

**Fig. 1:**
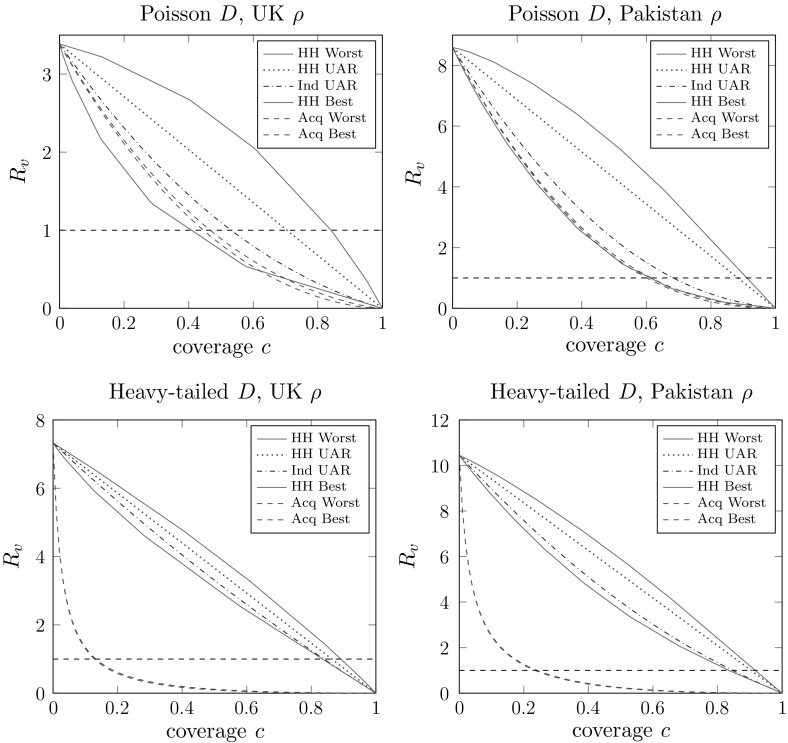
Plots of $R_v$ versus coverage (*c*) for various kinds of allocation of a perfect vaccine. We use two different household size distributions: *left plots* $\rho =\rho _{{\mathrm {UK}}}$, *right plots* $\rho =\rho _{{\mathrm {Pak}}}$; and two different degree distributions: *upper plots* $D\sim \text{ Poi }(5)$, *lower plots* $D\sim \text{ PowC }(2,120)$. Other parameters are $I\sim \text{ Gam }(5,1/5)$, $\lambda _L=1$, $\lambda _G=0.3$

First we look at the relative effectiveness of the various allocation schemes, and how this changes with the household size distribution and the network degree distribution. Figure 1 shows plots of the post-vaccination threshold parameter $R_v$ as a function of the vaccine coverage *c* for the 6 vaccine allocation schemes with a perfect vaccine. The different plots use different combinations of household size and network degree distribution, with all other parameters kept fixed (full details are in the figure caption). Firstly we note that the patterns of the household-based allocation schemes are consistent with those of Ball and Lyne (2006, Sect. 4) for the standard households model. We see that when the network degree distribution is not very variable (Poisson) good household-based schemes perform similarly to acquaintance vaccination. On the other hand, when the network degree distribution is much more variable (cut-off power law) we see that acquaintance vaccination significantly outperforms the household-based schemes, though to a slightly lesser extent when the household size distribution is also more variable.

**Fig. 2:**
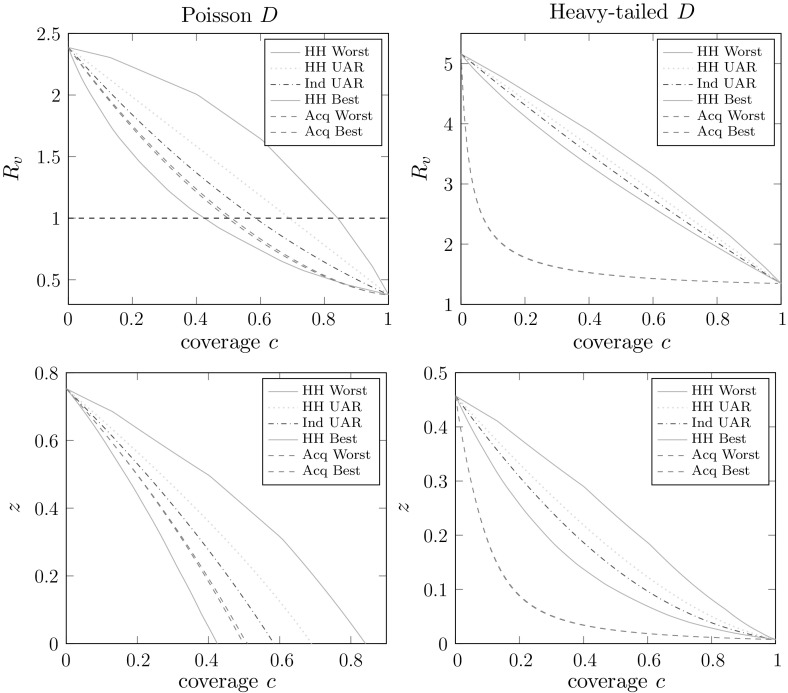
Plots of $R_v$ and *z* versus coverage (*c*) for various kinds of allocation of an all-or-nothing vaccine with success probability $\varepsilon =0.7$. We use two different degree distributions: *left plots* $D\sim \text{ Poi }(5)$, *right plots* $D\sim \text{ PowC }(2,120)$. Other parameters are $\rho =\rho _{{\mathrm {UK}}}$, $I\sim \text{ Gam }(5,1/5)$, $\lambda _L=1$, $\lambda _G=0.2$

**Fig. 3:**
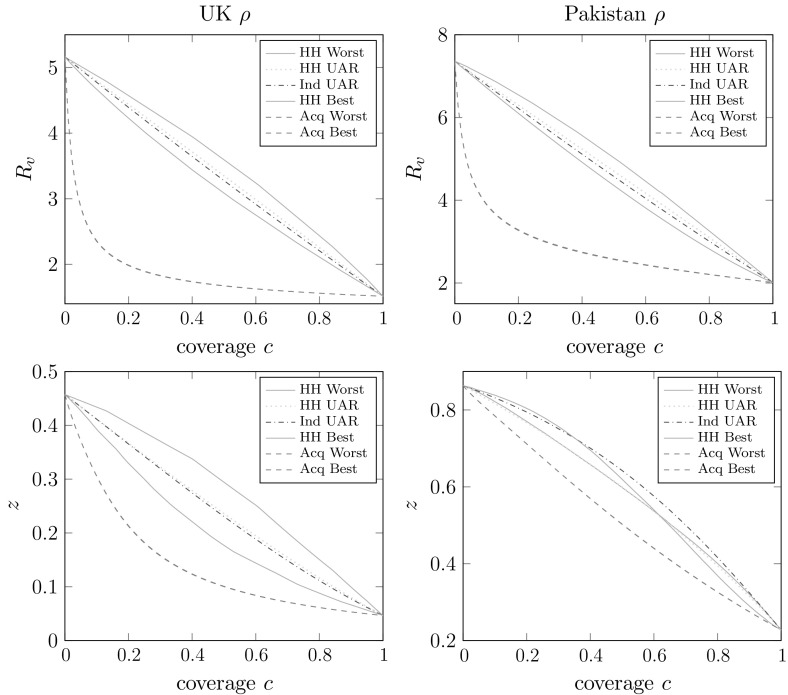
Plots of $R_v$ and *z* versus coverage (*c*) for various kinds of allocation of a non-random vaccine with relative susceptibility and infectivity $a=0.5$ and $b=0.6$ respectively. We use two different household size distributions: *left plots* $\rho =\rho _{{\mathrm {UK}}}$, *right plots* $\rho =\rho _{{\mathrm {Pak}}}$. Other parameters are $D\sim \text{ PowC }(2,120)$, $I\sim \text{ Gam }(5,1/5)$, $\lambda _L=1$, $\lambda _G=0.2$

Figures 2 and 3 are similar to Fig. 1, but we consider imperfect vaccine action models (with the same *efficacy* $1-E[AB]=0.7$) and we plot both the post-vaccination threshold parameter $R_v$ and expected final size of a large outbreak *z*. In Fig. 2 we use an all-or-nothing vaccine action model with success probability $\varepsilon =0.7$ and consider two network degree distributions, with fixed (less variable) household size distribution. In Fig. 3 we use a non-random vaccine action model with relative susceptibility and infectivity $a=0.5$ and $b=0.6$, respectively, and vary the household size distibution, with a fixed (more variable) network degree distribution. Note that for this choice of *a* and *b*, determining the ‘best’ and ‘worst’ household-based vaccine allocation schemes are not linear programming problems (see Sect. 3.4). In our numerical routine we use the MATLAB constrained optimisation solver fmincon.

We see in the upper plots (of $R_v$) in Figs. 2 and 3 broadly similar patterns to those in Fig. 1. The lower plots in Fig. 2 show the ordering of *z* for the different allocation regimes being the same as the ordering of $R_v$. These two lower plots look qualitatively quite different, but it is important to note that in one the vaccine can and in the other the vaccine cannot bring the epidemic below threshold. These plots suggest that, as might be expected, when the network degree distribution is not so variable (e.g. the Poisson case) then vaccine allocation should be focussed on households-based methods, whilst when the network degree distribution is more variable (e.g. the cutoff power law) then targeting vaccination effort based on the network might give better results. Precisely which method is preferable will of course depend heavily on the other parameters of the model, but we have demonstrated that either allocation method, household-based or acquaintance-based, can be preferable to the other.

The lower plots of Fig. 3 follow similar patterns in that we are considering the case of a quite variable network degree distribution so acquaintance vaccination outperforms the household-based methods. There are however some unexpected patterns in the lower-right plot of Fig. 3, in that the ordering of the various household-based allocation regimes are not the same as in the corresponding plot of $R_v$ immediately above it. In particular, for lower coverages the ‘worst’ households based allocation outperforms, in terms of *z*, the ‘best’! This demonstrates that when the epidemic is well above threshold, optimising vaccine allocation based on $R_*$ does not necessarily result in an expected final size that is as low as possible; cf. Keeling and Ross (2015), who observe a similar phenomenon in the standard households model with a perfect vaccine. The threshold parameter $R_v$ measures household-to-household transmission, but does not directly take into account the size of the within-household outbreaks. This could perhaps be resolved by optimising an individual-based threshold parameter instead (see Pellis et al. 2012; Ball et al. 2016), but the optimisation problem would be more difficult than the one we have considered. We also note that this phenomenon appears to arise only when the epidemic is well above criticality.

Lastly we note that the behaviour of $p_{{\mathrm {maj}}}$ is broadly similar to that of *z*. We do not present any plots, but they have shapes and patterns similar to those in the *z* plots that are shown, including the unexpected ordering observed in the lower-right plot of Fig. 3.

## Concluding remarks

In this paper we have analysed vaccine allocation strategies in stochastic SIR epidemic models upon populations with household and random network (of configuration model type) structure. By exploiting branching process approximations we derive asymptotic results describing the threshold behaviour of this epidemic model when there are few initial infectives and the final outcome in the event of a major outbreak. Particular attention has been paid to the analysis of acquaintance vaccination, which aims to target vaccination at individuals who are highly connected in the network. We find that acquaintance vaccination potentially offers substantial benefits over other (households-based) vaccine allocation regimes.

Whilst we have shown that acquaintance vaccination is potentially useful, it is clearly not feasible to implement in practice in human populations, so investigation of a more ethically acceptable allocation regime that preserves the targeting of well-connected individuals is a clear direction for future work. It is also likely that the effectiveness of acquaintance vaccination will depend on the amount of clustering in the network in an interesting way; we have touched on this through the use of different household size distributions, but clearly there is scope for considerably more work in this direction. Further issues that warrant more investigation include (i) comparison to the optimal network-based vaccine allocation, assuming knowledge of the degree of every individual in the network (Ball and Sirl 2012, Sect. 5 and Appendix B), (ii) the determination of optimal or near-optimal vaccination strategies based on household and network information and (iii) extending acquaintance vaccination to include the possiblity of naming individuals who are in the same household.

A particularly striking feature of the numerical study is the fact that, when vaccine coverage is insufficient to prevent a major outbreak, the ordering of the performance of the household-based allocation strategies can be different depending on whether $R_v$ (the post-vaccination $R_*$) or *z* is used as the measure of performance. We emphasise that this does not imply that minimising $R_v$ is not worthwhile. When using household-based vaccine allocation with a limited quantity of vaccine (i.e. a maximum vaccination coverage, *c* say), a natural approach is first to minimise $R_v$ among all strategies having coverage *c*. If the minimised value of $R_v$ is no greater than 1 then major outbreaks can be prevented and $R_v$ can be used to determine the vaccine allocation that prevents a major outbreak with minimum vaccination coverage. If the minimised value of $R_v$ is not less than 1 then households-based vaccination cannot prevent major outbreaks with the available quantity of vaccine. It then makes sense to minimise the expected cost of an outbreak; measured for example by $p_{{\mathrm {maj}}}$, *z* or some combination of these such as their product, which gives the probability that a typical individual is infected by the epidemic. The numerical examples in Sect. 5.3 suggest that in these circumstances minimising $R_v$ (which is a much simpler optimisation problem than, for example, minimising *z*) still produces a near-optimal vaccine allocation unless, post-vaccination, the epidemic is still well above threshold. However, this topic is not straightforward and clearly warrants further research. It will be investigated in more detail in a subsequent paper.

We also note in closing that there are some numerical challenges involved in implementing the methods we have presented. These are particularly relevant for the calculations relating to the forward process (i.e. calculation of $p_{{\mathrm {maj}}}$), but do also apply to the backward process (calculation of *z*). The main issue that arises is that of slowly converging infinite sums and the resulting possibilities for numerical overflow and underflow. Writing doubly or triply-infinite sums as one infinite and one or two finite sums (for example $\sum _{i=0}^\infty \sum _{j=0}^\infty a_{i,j}= \sum _{k=0}^\infty \sum _{l=0}^k a_{k-l,l}$) helps in some regards (e.g. faster computing since there is only one truncation to have to control the error of) but hinders in others (e.g. slower computing since methods to avoid underflow and overflow errors become more involved).

## Appendix A: Acquaintance vaccination branching process offspring distributions

In this appendix we consider the detail of calculating the offspring PGFs and means of the approximating branching processes for the acquaintance vaccination model described in Sect. 4.

In our analysis of the approximating branching processes (both forward and backward) it is convenient to think of the process of an individual in a household producing offspring (i.e. infecting network neighbours and thus contributing to the offspring of its household) in two phases. In the forward process the first phase involves consideration of the individual’s network neighbours, excluding its infector if it is a primary case. We consider how many such neighbours there are and what type they (and therefore their household) would be if they were to be infected. We call these individuals *next-generation neighbours*. In the second phase we consider which of these next-generation neighbours are actually infected and thus become primary cases in the offspring households. The above description of the first phase applies *mutatis mutandis* to the backward process; here the second phase is different in that the possible infectious contacts originate from outside rather than within the household under consideration. Since the first phase is the same in both the forward and backward branching processes we first consider it separately.

In Sect. A.1 we study the next-generation neighbour distributions. In Sect. A.2 we determine the mean offspring matrix for the forward process, and hence the post-vaccination threshold parameter $R_v$; in Sect. A.3 we consider the offspring distribution PGFs for the forward process, and hence the probability of a major outbreak; and in Sect. A.4 we determine the offspring PGFs for the backward process, and hence the relative final size of a major outbreak. The offspring distribution PGFs for the forward process are generally more complicated and some further detail of their calculation is described in Appendix B.4.

### A.1 Degree and next-generation neighbour distributions

As outlined above, the first phase of individuals producing offspring (which this subsection describes) applies equally to the forward and backward processes. To simplify the exposition we use only the language of the forward process of epidemic spread. We also use ‘household’ and ‘individual’ in the population sense (as opposed to the branching process sense where an individual corresponds to a household in the epidemic model).

Recall the typing described in Sect. 4.2, in which infectious households are characterised by the type ($1,2,\dots ,6$) of their primary case (see Table 1). Secondary cases in a household and the primary case in the initially infected household are typed (*V* or *U*) according to whether they are vaccinated or unvaccinated. Recall also that next-generation neighbours of an infected individual are the individuals (in neighbouring households) that it may infect. In this subsection we first calculate the degree distribution of the various secondary and primary cases conditional on their type. Then we consider the next-generation neighbour distributions: the number of network neighbours, excluding its infector, of each secondary type that a typical type-*i* individual has (for $i\in \{1,2,\dots ,6,U,V\}$).

#### A.1.1 Degree distributions

Let $D_V$ and $D_U$ respectively denote the degree of a typical vaccinated and unvaccinated secondary case. Then, recalling from Eq. (4.1) that $p_V=1-f_D(1-p_Sp_N)$,

A.1 $$\begin{aligned} P(D_U =d) = \frac{P(D=d)P(U|D=d)}{P(U)}=\frac{p_d (1-p_S p_N)^d}{1-p_V} \quad (d=0,1,\ldots ) \end{aligned}$$

and, similarly,

$$\begin{aligned} P(D_V=d)=\frac{p_d (1-(1-p_S p_N)^d)}{p_V} \quad (d=1,2,\ldots ). \end{aligned}$$

These follow because an unvaccinated individual has avoided being named by any of its neighbours and a vaccinated individual has not done so.

Now consider an individual of one of the primary types $1,2,\ldots ,6$. Note that whether or not it is sampled is independent of its degree. Thus primary cases of types 1 and 4 have the same degree distribution, as do primary cases of types 2 and 5, and primary cases of types 3 and 6. Let ${\tilde{D}}_N$, ${\tilde{D}}_V$ and ${\tilde{D}}_U$ denote generic random variables having these respective distributions. First note that ${\tilde{D}}_N \mathop {=}\limits ^{D} {\tilde{D}}$, where $\mathop {=}\limits ^{D}$ denotes equality in distribution. Second, consider a typical unvaccinated primary case. It has unconditional degree distribution ${\tilde{D}}$ but, in addition to not being named by its infector, we also know that it is not named by any of its other global neighbours. Thus

A.2 $$\begin{aligned} P({\tilde{D}}_U = d) = \frac{P({\tilde{D}}=d)P(U|{\tilde{D}}=d)}{1-{\tilde{p}}_V} = \frac{{\tilde{p}}_d (1-p_N p_S)^{d-1}}{1-{\tilde{p}}_V} \quad (d=1,2,\ldots ), \end{aligned}$$

where

A.3 $$\begin{aligned} {\tilde{p}}_V = \sum _{d=1}^{\infty } {\tilde{p}}_d (1-(1-p_N p_S)^{d-1}) = 1-f_{{\tilde{D}}-1} (1-p_N p_S) \end{aligned}$$

is the probability that a typical unnamed neighbour of an infector is vaccinated. Similarly,

A.4 $$\begin{aligned} P({\tilde{D}}_V=d)=\frac{{\tilde{p}}_d (1-(1-p_N p_S)^{d-1})}{{\tilde{p}}_V} \quad (d=2,3,\ldots ). \end{aligned}$$

#### A.1.2 Next-generation neighbour distributions

For $i=1,2,\dots ,6$ we write ${\tilde{{\varvec{X}}}}_i = ({\tilde{X}}_{i1}, {\tilde{X}}_{i2}, \dots ,{\tilde{X}}_{i6})$ for the number of next-generation neighbours of a type-*i* primary case; i.e. ${\tilde{X}}_{ij}$ is the number of neighbours of a type-*i* individual who will become type-*j* individuals if they are infected by the type-*i* primary case. For $A=U,V$ we similarly define ${\varvec{X}}_A = (X_{A1}, X_{A2}, \dots ,X_{A6})$ to be the corresponding quantities for a secondary case or the primary case in the initial household. To calculate the threshold parameter we need expressions for the means ${\hat{\mu }}_{ij}$ and ${\hat{\mu }}_{Aj}$ of the elements of these random vectors and to calculate the offspring distribution PGFs we require the joint PGFs $f_{{\tilde{{\varvec{X}}}}_i}$ and $f_{{\varvec{X}}_A}$.

*Means*   The mean $E[{\tilde{X}}_{ij}]={\hat{\mu }}_{ij}$ can be written as ${\hat{\mu }}_{ij} = {\tilde{\mu }}^G_i {\hat{p}}_{ij}$, where ${\tilde{\mu }}_i^G$ is the mean number of network neighbours a typical type-*i* primary case has in addition to its infector and ${\hat{p}}_{ij}$ is the probability that a given such global neighbour is of type *j* (or, more accurately, the probability it will be of type *j* if it is infected). Similarly we write $E[X_{Aj}] = {\hat{\mu }}_{Aj} = \mu ^G_A {\hat{p}}_{Aj}$ for the secondary cases and primary case in the initial household.

Consider a type-1 (i.e. (*N*, *S*)) primary case. Its global degree is distributed as ${\tilde{D}}_N \mathop {=}\limits ^{D} {\tilde{D}}$, so ${\tilde{\mu }}_1^G = \mu _{{\tilde{D}}-1}$. A given neighbour is named with probability $p_N$, and, if that neighbour is unnamed, it is vaccinated with probability ${\tilde{p}}_V$ and otherwise unvaccinated. Further, independently, that neighbour is sampled with probability $p_S$. So, ${\hat{p}}_{11} = p_N p_S$, ${\hat{p}}_{12} = (1-p_N) {\tilde{p}}_V p_S$, ${\hat{p}}_{13} = (1-p_N)(1-{\tilde{p}}_V)p_S$, ${\hat{p}}_{14}=p_N (1-p_S)$, ${\hat{p}}_{15} = (1-p_N) {\tilde{p}}_V (1-p_S)$ and ${\hat{p}}_{16} = (1-p_N) (1-{\tilde{p}}_V )(1-p_S)$. The situation is similar for a type-4 (i.e. $(N,S^c)$) individual, except a type-4 individual is not sampled and hence cannot name its global neighbours. Hence ${\tilde{\mu }}_4^G = \mu _{{\tilde{D}}-1}$, ${\hat{p}}_{41} = {\hat{p}}_{44}=0$, ${\hat{p}}_{42} = {\tilde{p}}_V p_S$, ${\hat{p}}_{43} = (1-{\tilde{p}}_V) p_S$, ${\hat{p}}_{45} = {\tilde{p}}_V (1-p_S)$ and ${\hat{p}}_{46} = (1-{\tilde{p}}_V)(1-p_S)$.

Next consider a type-2 (i.e. (*V*, *S*)) primary case, $i^{*}$ say. Its global degree is distributed as ${\tilde{D}}_V$, so ${\tilde{\mu }}_2^G = \mu _{{\tilde{D}}_V -1}$ and, using (A.4),

$$\begin{aligned} \mu _{{\tilde{D}}_V -1} = \frac{\mu _{{\tilde{D}}-1} - (1-p_N p_S) f'_{{\tilde{D}}-1} (1-p_N p_S)}{{\tilde{p}}_V}. \end{aligned}$$

Let ${\tilde{N}}_S$ be the number of next-generation neighbours of $i^{*}$ that are sampled. Then ${\tilde{N}}_S \ge 1$, since $i^{*}$ is vaccinated but not named by its global infector, and, for $k=1,2,\ldots ,d-1$,

A.5 $$\begin{aligned} P({\tilde{N}}_S= & {} k \mid {\tilde{D}}_V = d) = \frac{\left( {\begin{array}{c}d-1\\ k\end{array}}\right) p_S^k (1-p_S)^{d-1-k} (1-(1-p_N)^k)}{1-(1-p_N p_S)^{d-1}}, \end{aligned}$$

whence, using (A.4),

A.6 $$\begin{aligned} \mu _{{\tilde{N}}_S}= \frac{p_S \left( \mu _{{\tilde{D}}-1} - (1-p_N) f'_{{\tilde{D}}-1}(1-p_N p_S )\right) }{{\tilde{p}}_V}. \end{aligned}$$

It follows that the probability that a given next-generation neighbour of $i^{*}$ is sampled is $p_S^{{\tilde{V}}} = \mu _{{\tilde{N}}_S} / {\tilde{\mu }}_2^G$ and that, for $j=1,2,\ldots ,6$, ${\hat{p}}_{2j}$ is given by ${\hat{p}}_{1j}$ with $p_S$ replaced by $p_S^{{\tilde{V}}}$. Similar arguments for a type-5 (i.e. $(U,S^c)$) primary case establish that ${\tilde{\mu }}_5^G = \mu _{{\tilde{D}}_V -1}$ and, for $j=1,2,\ldots ,6$, ${\hat{p}}_{5j}$ is given by ${\hat{p}}_{4j}$ with $p_S$ replaced by $p_S^{{\tilde{V}}}$.

Now consider a type-3 (i.e. (*U*, *S*)) primary case, $j^{*}$ say. Its global degree is distributed as ${\tilde{D}}_U$, so ${\tilde{\mu }}_3^G = \mu _{{\tilde{D}}_U-1}$ and, using (A.2),

A.7 $$\begin{aligned} \mu _{{\tilde{D}}_U-1} = \frac{(1-p_N p_S) f'_{{\tilde{D}}-1} (1-p_N p_S)}{1-{\tilde{p}}_V}. \end{aligned}$$

Since $j^{*}$ is not vaccinated, none of its next-generation neighbours name $j^{*}$, so the probability that a given such neighbour, $k^{*}$ say, is sampled is given by

$$\begin{aligned} p_S^U = P(k^{*} \text { sampled} \mid k^{*} \text { does not name } j^*)=\frac{p_S (1-p_N)}{1-p_S p_N}. \end{aligned}$$

It follows that, for $j=1,2,\ldots ,6$, ${\hat{p}}_{3j}$ is given by ${\hat{p}}_{1j}$ with $p_S$ replaced by $p_S^U$. Similarly, ${\tilde{\mu }}_6^G = \mu _{{\tilde{D}}_U-1}$ and, for $j=1,2,\ldots ,6$, ${\hat{p}}_{6j}$ is given by ${\hat{p}}_{4j}$ with $p_S$ replaced by $p_S^U$.

Now consider an individual, $i^*$ say, of type *U* (i.e. chosen uniformly from the population and unvaccinated). Its global degree is distributed as $D_U$ so, using (A.1), its expected number of next-generation neighbours is

A.8 $$\begin{aligned} \mu ^G_U=\mu _{D_U} = \frac{(1-p_S p_N) f'_D (1-p_S p_N)}{1-p_V}. \end{aligned}$$

Further, $i^*$ is sampled with probability $p_S$, and the type of a given neighbour of $i^{*}$ is distributed according to ${\hat{p}}_{3j}$ if $i^{*}$ is sampled and according to ${\hat{p}}_{6j}$ if $i^{*}$ is not sampled. Thus ${\hat{p}}_{Uj}=p_S {\hat{p}}_{3j} + (1-p_S) {\hat{p}}_{6j}$.

Finally, consider an individual, $j^{*}$ say, of type *V* (i.e. chosen uniformly from the population and vaccinated). Let $N_S$ and $N_{S^c}$ denote the number of next-generation neighbours of $j^{*}$ that are sampled and not sampled, respectively. Then arguing as in the derivation of (A.6) shows that

$$\begin{aligned} \mu _{N_S} = \frac{p_S \left( \mu _D - (1-p_N) f'_D (1-p_S p_N)\right) }{p_V} \end{aligned}$$

and

$$\begin{aligned} \mu _{N_{S^c}} = \frac{(1-p_S)\left( \mu _D - f'_D (1-p_S p_N)\right) }{p_V}. \end{aligned}$$

Further, $j^{*}$ is sampled with probability $p_S$. If $j^{*}$ is sampled then a given neighbour is named, vaccinated or unvaccinated with respective probabilities $p_N$, $(1-p_N) {\tilde{p}}_V$ and $(1-p_N)(1-{\tilde{p}}_V)$; whilst if $j^{*}$ is not sampled these probabilities are 0, ${\tilde{p}}_V$ and $1-{\tilde{p}}_V$. We therefore have ${\hat{p}}_{Vj} = p_S {\hat{p}}_{1j} + (1-p_S) {\hat{p}}_{4j}$.

*Joint PGFs*   We determine next the PGFs $f_{{\tilde{{\varvec{X}}}}_i}$ ($i=1,2,\ldots ,6$). For $i=1,2,\ldots ,6$, let ${\tilde{D}}(i)$ denote the degree of a typical type-*i* primary individual, who thus has ${\tilde{D}}(i)-1$ next-generation neighbours. We therefore have ${\tilde{D}}(i) -1 \mathop {=}\limits ^{D} \sum _{j=1}^6 {\tilde{X}}_{ij}$. For $i=1,3,4$ and 6, the types of these ${\tilde{D}}(i)-1$ next-generation neighbours are chosen independently, according to $({\hat{p}}_{ij},\, j=1,2,\ldots ,6)$, so

$$\begin{aligned} f_{{\tilde{{\varvec{X}}}}_i} ({\varvec{s}}) = f_{{\tilde{D}}(i)-1} ({\hat{g}}_i ({\varvec{s}})) \quad (i=1,3,4,6), \end{aligned}$$

where ${\hat{g}}_i ({\varvec{s}}) = \sum _{j=1}^6 {\hat{p}}_{ij} s_j$. Further ${\tilde{D}}(1) \mathop {=}\limits ^{D} {\tilde{D}}(4) \sim {\tilde{D}}$ and ${\tilde{D}}(3) \mathop {=}\limits ^{D} {\tilde{D}}(6) \sim {\tilde{D}}_U$ [see (A.2)].

Now consider a typical type-2 primary individual. The situation is more complex here (and for type-5 individuals) because we know that at least one of its neighbours named it for vaccination and thus must be sampled. Let ${\tilde{N}}_S$ and ${\tilde{N}}_{S^c}$ be the number of its ${\tilde{D}}(2)-1$ next-generation neighbours that are sampled and unsampled, respectively, so ${\tilde{N}}_S + {\tilde{N}}_{S^c}={\tilde{D}}(2)-1$. Each of these ${\tilde{D}}(2)-1$ neighbours is named independently with probability $p_N$ and any neighbour not so named is vaccinated with probability ${\tilde{p}}_V$. Thus

$$\begin{aligned} f_{{\tilde{{\varvec{X}}}}_2} ({\varvec{s}}) = E \left[ ({\hat{g}}_{21} ({\varvec{s}}))^{{\tilde{N}}_S} ({\hat{g}}_{22} ({\varvec{s}}))^{{\tilde{N}}_{S^c}}\right] , \end{aligned}$$

where ${\hat{g}}_{21} ({\varvec{s}}) = p_N s_1 + (1-p_N) {\tilde{p}}_V s_2 + (1-p_N )(1-{\tilde{p}}_V) s_3$ and ${\hat{g}}_{22} ({\varvec{s}}) = p_N s_4 + (1-p_N) {\tilde{p}}_V s_5 + (1-p_N) (1-{\tilde{p}}_V)s_6$. Furthermore, ${\tilde{D}}(2) \sim {\tilde{D}}_V$, whence using (A.4) and (A.5) we obtain

A.9 $$\begin{aligned} f_{{\tilde{{\varvec{X}}}}_2} ({\varvec{s}}) = {\tilde{p}}_V^{-1} \left[ f_{{\tilde{D}}-1} \left( {\hat{g}}_2 ({\varvec{s}}, 0) \right) - f_{{\tilde{D}}-1} \left( {\hat{g}}_2 ({\varvec{s}}, p_N )\right) \right] , \end{aligned}$$

where ${\hat{g}}_2 ({\varvec{s}},x)=p_S (1-x) {\hat{g}}_{21} ({\varvec{s}}) + (1-p_S) {\hat{g}}_{22} ({\varvec{s}})$. A similar argument shows that

A.10 $$\begin{aligned} f_{{\tilde{{\varvec{X}}}}_5} ({\varvec{s}}) = {\tilde{p}}_V^{-1} \left[ f_{{\tilde{D}}-1} \left( {\hat{g}}_5 ({\varvec{s}},0) \right) - f_{{\tilde{D}}-1} \left( {\hat{g}}_5 ({\varvec{s}},p_N) \right) \right] , \end{aligned}$$

where ${\hat{g}}_5 ({\varvec{s}},x) = p_S (1-x) {\hat{g}}_{51} ({\varvec{s}}) + (1-p_S) {\hat{g}}_{52} ({\varvec{s}})$, with ${\hat{g}}_{51} ({\varvec{s}}) = {\tilde{p}}_V s_2 + (1-{\tilde{p}}_V) s_3$ and ${\hat{g}}_{52} ({\varvec{s}}) = {\tilde{p}}_V s_5 + (1-{\tilde{p}}_V)s_6$.

A typical type-*U* secondary individual, $j_U^{*}$ say, has degree distributed according to $D_U$ [recall (A.1)], so its number of next-generation neighbours is also distributed as $D_U$. Further, $j_U^{*}$ is sampled with probability $p_S$, in which case, apart from its degree, $j_U^{*}$ behaves similarly to a type-3 primary individual, otherwise $j_U^{*}$ is not sampled and behaves similarly to a type-6 primary individual. Thus,

$$\begin{aligned} f_{{\varvec{X}}_U} ({\varvec{s}}) = p_S f_{D_U} ({\hat{g}}_3 ({\varvec{s}})) + (1-p_S) f_{D_U} ({\hat{g}}_6 ({\varvec{s}})). \end{aligned}$$

A typical type-*V* secondary individual, $j_V^{*}$ say, is also sampled with probability $p_S$. A similar argument to the derivation of (A.9) and (A.10), using appropriate modifications as $j_V^{*}$ has degree distributed according to $D_V$, yields

$$\begin{aligned} f_{{\varvec{X}}_V} ({\varvec{s}})&= p_V^{-1} \left\{ p_S \left[ f_D \left( {\hat{g}}_2 ({\varvec{s}},0)\right) - f_D \left( {\hat{g}}_2 ({\varvec{s}},p_N)\right) \right] \right. \\&\qquad \qquad +\left. (1-p_S)\left[ f_D\left( {\hat{g}}_5 ({\varvec{s}},0)\right) - f_D\left( {\hat{g}}_5 ({\varvec{s}},p_N)\right) \right] \right\} . \end{aligned}$$

### A.2 Threshold parameter

As far as is possible, we treat the all-or-nothing and non-random models simultaneously. As explained in the second paragraph of Sect. 4.2.2, in contrast to Sect. 3.2, when analysing the all-or-nothing vaccine response we consider actual, rather than potential, global infections. The forward branching process for both models is then similar to that used for the non-random model in Sect. 3.3, except now it is a 6-type (rather than 2-type) branching process. As always, the offspring distributions are different in the initial generation from subsequent generations, but only those for the latter are required to determine the threshold parameter. Recall that, for $i=1,2,\ldots ,6$, ${\tilde{{\varvec{C}}}}_i = ({\tilde{C}}_{i1}, {\tilde{C}}_{i2}, \ldots , {\tilde{C}}_{i6})$ denotes the offspring random variable for a type-*i* non-initial individual in the forward branching process. Thus ${\tilde{C}}_{ij}$ is the number of type-*j* primary household cases emanating from a typical single-household epidemic that is initiated by a single type-*i* primary case. Since the post-vaccination threshold parameter $R_v$ is the dominant eigenvalue of ${\tilde{M}}=[{\tilde{m}}_{ij}]$, our goal is to determine these matrix entries ${\tilde{m}}_{ij}=E[{\tilde{C}}_{ij}]$.

To calculate ${\tilde{m}}_{ij}$ it is convenient to decompose it into ${\tilde{m}}_{ij} = {\tilde{m}}_{ij}^P + {\tilde{m}}_{ij}^S$, where ${\tilde{m}}_{ij}^P$ is the mean number of global infections of type-*j* individuals emanating from the primary type-*i* case and ${\tilde{m}}_{ij}^S$ is the mean total number of global infections of type-*j* individuals emanating from all secondary cases in the single-household epidemic.

#### A.2.1 Infections emamating from primary cases

Define the matrix $P_G$ of marginal global transmission probabilities, so $P_G = P_G^{\mathrm{NR}}$ [recall (3.7)] if the vaccine action is non-random and $P_G = P_G^\mathrm{AoN}$ if the vaccine action is all-or-nothing, where

$$\begin{aligned} P_G^\mathrm{AoN} = \left[ \begin{array}{ll} p_G^\mathrm{AoN} (U,U)&{}\quad p_G^\mathrm{AoN} (U,V)\\ p_G^\mathrm{AoN} (V,U)&{}\quad p_G^\mathrm{AoN} (V,V)\end{array}\right] = (1-\phi _I(\lambda _G)) \left[ \begin{array}{ll}1&{}\quad 1-\varepsilon \\ 1&{}\quad 1-\varepsilon \end{array}\right] . \end{aligned}$$

The marginal global transmission probabilities, for the types $i,j = 1,2,\ldots ,6$, are then given by $p_{ij}^G = p_G (\alpha (i),\alpha (j))$, where $\alpha (k) = U$ if $k=3,6$ and $\alpha (k)=V$ if $k=1,2,4,5$. Since each type-*j* next-generation neighbour of a type-*i* infective is infected with probability $p_{ij}^G$ it is immediate that ${\tilde{m}}_{ij}^P = {\hat{\mu }}_{ij} p_{ij}^G$ , where ${\hat{\mu }}_{ij}$ is the mean number of next-generation neighbours calculated in Sect. A.1.2.

#### A.2.2 Infections emamating from secondary cases

We first note that, for $j=1,2,\ldots ,6$, ${\tilde{m}}_{1j}^S = {\tilde{m}}_{2j}^S = {\tilde{m}}_{4j}^S = {\tilde{m}}_{5j}^S$ ($=\,{\tilde{m}}_{Vj}^S$ say) and ${\tilde{m}}_{3j}^S = {\tilde{m}}_{6j}^S$ ($=$ ${\tilde{m}}_{Uj}^S$ say). Further, for $A \in \{ U,V \}$ and $j=1,2,\ldots ,6$,

$$\begin{aligned} {\tilde{m}}_{Aj}^S = \mu _S (A,U) {\hat{m}}_{Uj} + \mu _S (A,V) {\hat{m}}_{Vj}, \end{aligned}$$

where, for $A,A' \in \{U,V\}$, $\mu _S (A,A')$ is the mean number of type-$A'$ secondary cases in a typical single-household epidemic initiated by a primary case of type *A* and, for $A \in \{ U,V \}$, ${\hat{m}}_{Aj}$ is the mean number of type-*j* primary cases emanating from a typical type-*A* secondary case.

Now observe that ${\hat{m}}_{Aj}={\hat{\mu }}_{Aj}p_G(A,\alpha (j))$, where $\alpha (j)$ is as in Sect. A.2.1. Conditioning on the size of a typical globally contacted household, we obtain in an obvious notation that

$$\begin{aligned} \mu _S (A,A') = \sum _{n=1}^{\infty } {\tilde{\rho }}_n \mu _S^{(n)} (A,A') \quad (A,A' \in \{U,V\}). \end{aligned}$$

Note that in the limit as the number of households $m\rightarrow \infty$, secondary individuals in a typical household have no common global neighbour with probability one, so they are vaccinated independently, each with probability $p_V$. Thus, again in an obvious notation,

$$\begin{aligned} \mu _S^{(n)} (A,A') = \sum _{k=0}^{n-1} \left( {\begin{array}{c}n-1\\ k\end{array}}\right) p_V^k (1-p_V)^{n-1-k} \mu _S^{(n,k)} (A,A'). \end{aligned}$$

If the vaccine action is non-random then, recalling the notation in Sect. 3.3, $\mu _S^{(n,k)} (A,A') = \mu ^{(n,k+\delta _{A,V})} (A,A')$, where $\delta _{A,V}=1$ if $A=V$ and 0 otherwise. If the vaccine action is all-or-nothing, we condition on the number of successfully vaccinated secondary individuals in the household to obtain, for $A \in \{ U,V \}$,

$$\begin{aligned} \mu _S^{(n,k)} (A,V) = \sum _{l=0}^{\min (k,n-2)} \left( {\begin{array}{c}k\\ l\end{array}}\right) \varepsilon ^l (1-\varepsilon ) ^{k-l} \mu ^{(n-l)} (\lambda _L) \left( \frac{k-l}{n-l-1}\right) \end{aligned}$$

and

$$\begin{aligned} \mu _S^{(n,k)} (A,U) = \sum _{l=0}^{\min (k,n-2)} \left( {\begin{array}{c}k\\ l\end{array}}\right) \varepsilon ^l (1-\varepsilon )^{k-l} \mu ^{(n-l)} (\lambda _L) \left( \frac{n-k-1}{n-l-1} \right) . \end{aligned}$$

(Note that $\mu ^{(n-l)}(\lambda _L)=0$ when $l=n-1$.)

#### A.2.3 Perfect vaccine

In the situation where the vaccine is perfect, we have only households of types 3 and 6 (i.e. (*U*, *S*) and $(U,S^c)$); and since these two types are sampled independently with the same probability, the forward process reduces to single-type. The mean number of next-generation neighbours of an infected individual is $\mu _{{\tilde{D}}_U-1} + \mu _S(U,U)\mu _{D_U}$. Each infected individual is sampled with probability $p_S^U$, so fails to name each next-generation neighbour with probability $p_S^U(1-p_N) + 1-p_S^U$. Then each such unnamed individual avoids vaccination (by other neighbours in the network) with probability $1-{\tilde{p}}_V$. Thus

$$\begin{aligned} R_v = (\mu _{{\tilde{D}}_U-1} + \mu _{D_U}\mu _S(U,U)) (p_S^U(1-p_N) + 1-p_S^U) (1-{\tilde{p}}_V) p_G. \end{aligned}$$

Using (A.3), (A.7) and (A.8) this can be written as

$$\begin{aligned} R_v =&\, f_{{\tilde{D}}-1}(1-p_Sp_N) p_G \left( \frac{f'_{{\tilde{D}}-1}(1-p_Sp_N)}{f_{{\tilde{D}}-1}(1-p_Sp_N)} + \mu _S(U,U)\frac{f'_D(1-p_Sp_N)}{f_D(1-p_Sp_N)}\right) \\&\qquad \times (1-2p_Sp_N+p_Sp_N^2)\\ =&\, \eta (p') (1-2p'+p'p_N), \end{aligned}$$

where $p'=p_Sp_N$ and $\eta$ depends on $p_S$ and $p_N$ only through $p'$.

### A.3 Probability of a major outbreak

To determine the probability of a major outbreak we need to derive the PGFs $f_{{\tilde{{\varvec{C}}}}_i} ({\varvec{s}})$ and $f_{{\varvec{C}}_A} ({\varvec{s}})$, where ${\varvec{s}}=(s_1,s_2,\ldots ,s_6) \in [0,1]^6$, for $i=1,2,\ldots ,6$ and $A=U,V$; i.e. the offspring PGFs for the forward process.

Recall that secondary individuals in a household are vaccinated independently, each with probability $p_V$. Thus, conditioning first on household size and then on the number of secondary members that are vaccinated yields, for $i=1,2,\ldots ,6$,

$$\begin{aligned} f_{{\tilde{{\varvec{C}}}}_i} ({\varvec{s}}) = \sum _{n=1}^{\infty } {\tilde{\rho }}_n \sum _{v_s=0}^{n-1} \left( {\begin{array}{c}n-1\\ v_s\end{array}}\right) p_V^{v_s} (1-p_V)^{n-1-v_s} f_{ {\tilde{{\varvec{C}}}}_i^{(n,v_s)}} ({\varvec{s}}), \end{aligned}$$

where ${\tilde{{\varvec{C}}}}_i^{(n,v_s)}$ is the offspring random variable for a type-*i* non-initial individual, given that the corresponding household is of size *n* and has $v_s$ secondary members vaccinated. Similarly, and in an obvious notation, for $A \in \{ U,V \}$,

$$\begin{aligned} f_{{\varvec{C}}_A} ({\varvec{s}}) = \sum _{n=1}^{\infty } {\tilde{\rho }}_n \sum _{v_s =0}^{n-1} \left( {\begin{array}{c}n-1\\ v_s\end{array}}\right) p_V^{v_s} (1-p_V)^{n-1-v_s} f_{{\varvec{C}}_A^{(n,v_s)}} ({\varvec{s}}). \end{aligned}$$

With an all-or-nothing vaccine action, we may condition also on the number of vaccinations of secondary members that are unsuccessful to obtain, for $i=1,2,\ldots ,6$,

$$\begin{aligned} f_{{\tilde{{\varvec{C}}}}_i^{(n,v_s)}}({\varvec{s}})=\sum _{u_s=0}^{v_s} \left( {\begin{array}{c}v_s\\ u_s\end{array}}\right) (1-\varepsilon )^{u_s} \varepsilon ^{v_s-u_s} f_{{\hat{{\varvec{C}}}}_i^{(n-v_s+u_s,u_s)}} ({\varvec{s}}), \end{aligned}$$

where ${\hat{{\varvec{C}}}}_i^{(n',v')}$ is the offspring random variable for a type-*i* non-initial individual, given that the corresponding household contains $n'-1$ other susceptibles, of which $v'$ are unsuccessfully vaccinated. (Note that, unlike with household-based vaccination, the vaccine status of these susceptibles is important as it affects their degree distributions.) Similarly, and in an obvious notation, for $A \in \{ U,V \}$,

$$\begin{aligned} f_{{\varvec{C}}_A^{(n,v_s)}}({\varvec{s}}) = \sum _{u_s=0}^{v_s} \left( {\begin{array}{c}v_s\\ u_s\end{array}}\right) (1-\varepsilon )^{u_s} \varepsilon ^{v_s-u_s} f_{{\hat{{\varvec{C}}}}_A^{(n-v_s+u_s,u_s)}} ({\varvec{s}}). \end{aligned}$$

The PGFs $f_{{\tilde{{\varvec{C}}}}_i^{(n,v_s)}} ({\varvec{s}})$ and $f_{{\varvec{C}}_A^{(n,v_s)}} ({\varvec{s}})$ when the vaccine action is non-random and $f_{{\hat{{\varvec{C}}}}_i^{(n',v')}}({\varvec{s}})$ and $f_{{\hat{{\varvec{C}}}}_A^{(n',v')}} ({\varvec{s}})$ when the vaccine action is all-or-nothing can be calculated using the methods described in Appendices B.1 and B.4. As with other calculations of PGFs relating to forward branching process approximations, these simplify appreciably if the infectious period is constant.

### A.4 Final outcome of major outbreak

To calculate the asymptotic final size of a major outbreak we need the joint PGFs $f_{{\tilde{{\varvec{B}}}}_i} ({\varvec{s}})$, for $i=1,2,\ldots ,6$, and $f_{{\varvec{B}}_A} ({\varvec{s}})$, for $A=U,V$; where ${\varvec{s}}=(s_1,s_2,\ldots ,s_6) \in [0,1]^6$. We determine first the former joint PGFs. For $i=1,2,\dots ,6$, let ${\varvec{M}}_i = (M_{iU}, M_{iV})$, where $M_{iU}$ and $M_{iV}$ are respectively the number of unvaccinated and vaccinated secondary individuals in the local susceptibility set of a typical type-*i* primary individual. Then ${\tilde{{\varvec{B}}}}_i$ admits the decomposition

A.11 $$\begin{aligned} {\tilde{{\varvec{B}}}}_i = {\hat{{\varvec{B}}}}_i^P + \sum _{k=1}^{M_{iU}} {\hat{{\varvec{B}}}}_U^S (k) + \sum _{l=1}^{M_{iV}} {\hat{{\varvec{B}}}}_V^S (l), \end{aligned}$$

where ${\hat{{\varvec{B}}}}_i^P$, ${\hat{{\varvec{B}}}}_U^S (k)$ and ${\hat{{\varvec{B}}}}_V^S (l)$ are the respective contributions to ${\tilde{{\varvec{B}}}}_i$ from the primary individual, the *k*th unvaccinated secondary individual and the *l*th vaccinated secondary individual in the local susceptibility set. All summands on the right hand side of (A.11) are mutually independent and also independent of ${\varvec{M}}_i$, so, in an obvious notation,

A.12 $$\begin{aligned} f_{{\tilde{{\varvec{B}}}}_i} ({\varvec{s}}) = f_{{\hat{{\varvec{B}}}}_i^P}({\varvec{s}}) f_{{\varvec{M}}_i} (f_{{\hat{{\varvec{B}}}}_U^S} ({\varvec{s}}), f_{{\hat{{\varvec{B}}}}_V^S} ({\varvec{s}})). \end{aligned}$$

To derive the PGF $f_{{\varvec{M}}_i}$, note that $f_{{\varvec{M}}_1}=f_{{\varvec{M}}_2} = f_{{\varvec{M}}_4} = f_{{\varvec{M}}_5}$ ($=f_{{\varvec{M}}_V}$ say) and $f_{{\varvec{M}}_3} = f_{{\varvec{M}}_6}$ ($=f_{{\varvec{M}}_U}$ say). Further, conditioning on the size of the household and recalling that secondary individuals in the household are vaccinated independently, each with probability $p_V$, yields

$$\begin{aligned} f_{{\varvec{M}}_A} ({\varvec{s}}) = \sum _{n=1}^{\infty } {\tilde{\rho }}_n \sum _{v=0}^{n-1} \left( {\begin{array}{c}n-1\\ v\end{array}}\right) p_V^v (1-p_V)^{n-1-v} f_{{\varvec{M}}_A^{(n,v+\delta _{A,V})}} ({\varvec{s}}) \quad ({\varvec{s}}\in [0,1]^2), \end{aligned}$$

for $A \in \{ U,V \}$, where ${\varvec{M}}_A^{(n,v)}$ is as in Sect. 3.3. Calculation of $f_{{\varvec{M}}_A^{(n,v)}} ({\varvec{s}})$ for the two models of vaccine action is addressed in Appendix B.5.

We determine next the PGFs $f_{{\hat{{\varvec{B}}}}_i^P}$ ($i=1,2,\ldots ,6$). We do so by conditioning on ${\tilde{{\varvec{X}}}}_i$, the numbers of next-generation neighbours of different types of a typical type-*i* primary case, $i^*$ say, and then considering how many of the ${\tilde{{\varvec{X}}}}_i$ individuals of the various types actually join the susceptibility set. Each of $i^*$’s ${\tilde{D}}(i)-1=\sum _{j=1}^6 {\tilde{X}}_{ij}$ next-generation neighbours in the construction of the backward process enters the susceptibility set independently, with probability depending on the types of the two individuals involved. The probability that a type-*j* neighbour of a type-*i* individual joins the susceptibility set is

$$\begin{aligned} p_{ij}^B = {\left\{ \begin{array}{ll} p_{ji}^G &{} \,\, \text{ non-random } \text{ vaccine, } \\ p_{ij}^G &{} \,\, \text{ all-or-nothing } \text{ vaccine. } \end{array}\right. } \end{aligned}$$

(The different formulae arise from the fact that in the all-or-nothing case, in addition to requiring a contact from the neighbour to the type-*i* individual of interest, a vaccinated neighbour is able to join the susceptibility set only if its vaccination fails.) Hence, for $i=1,2,\ldots ,6$,

$$\begin{aligned} f_{{\hat{{\varvec{B}}}}_i^P} ({\varvec{s}}) = f_{{\tilde{{\varvec{X}}}}_i} ({\varvec{h}}_i^B ({\varvec{s}})), \end{aligned}$$

where ${\varvec{h}}_i^B ({\varvec{s}}) = (h_{i1}^B (s_1), h_{i2}^B (s_2), \ldots , h_{i6}^B (s_6))$, with $h_{ij}^B (s) = 1-p^B_{ij} + sp^B_{ij}$ ($j=1,2,\ldots ,6$). The PGFs $f_{{\hat{{\varvec{B}}}}_U^S}$ and $f_{{\hat{{\varvec{B}}}}_V^S}$ follow using exactly the same arguments. In an obvious notation,

$$\begin{aligned} f_{{\hat{{\varvec{B}}}}_A^S} ({\varvec{s}}) = f_{{\varvec{X}}_A} ({\varvec{h}}_A^B ({\varvec{s}})) \quad (A \in \{U,V\}), \end{aligned}$$

where $${\varvec{h}}_U^B = {\varvec{h}}_3^B$$ and $${\varvec{h}}_V^B = {\varvec{h}}_1^B$$.

Finally, we determine the offspring PGFs for the initial generation, $f_{{\varvec{B}}_U}$ and $f_{{\varvec{B}}_V}$. Observe that, for $A \in \{ U,V \}$, in the initial generation, a primary individual of type *A* behaves according to the same probability law as a typical secondary type-*A* individual (in any generation), so (A.12) becomes

$$\begin{aligned} f_{{\varvec{B}}_A} ({\varvec{s}}) = f_{{\hat{{\varvec{B}}}}_A^S} ({\varvec{s}}) f_{{\varvec{M}}_A} ( f_{{\hat{{\varvec{B}}}}_U^S} ({\varvec{s}}), f_{{\hat{{\varvec{B}}}}_V^S} ({\varvec{s}}) ) \quad (A \in \{ U,V \}) \end{aligned}$$

and $f_{{\varvec{B}}_A} ({\varvec{s}})$ can be evaluated using results given above.

## Appendix B: Properties of single-household epidemics

In this appendix we first outline the theory of final state random variables as studied by Ball and O’Neill (1999). We then apply this (in Sects. B.2, B.3, B.4) to calculating the PGFs of offspring distributions of the various forward branching process approximations in the paper. In Sects. B.5 and B.6 we give results pertaining to the size of local susceptibility sets and the expected size of local epidemics.

We use the following notation. The symbols $\mathbb {Z}_+$ and $\mathbb {R}_+$ denote the non-negative integers and reals, respectively. For vectors ${\varvec{x}}=(x_1,x_2,\ldots ,x_m)$ and ${\varvec{y}}=(y_1,y_2,\ldots ,y_m)$ in $\mathbb {R}^m$, we define ${\varvec{x}}{\varvec{y}}=\prod _{i=1}^m x_i y_i$ and ${\varvec{x}}^{{\varvec{y}}}=\prod _{i=1}^m x_i^{y_i}$. We write ${\varvec{x}}\le {\varvec{y}}$ if $x_i \le y_i$ $(i=1,2,\ldots ,m)$ and ${\varvec{x}}<{\varvec{y}}$ if, in addition, $x_i<y_i$ for at least one *i*. For $n,k \in \mathbb {Z}_+, n_{[k]}$ denotes the falling factorial $n(n-1)\ldots (n-k+1)$, with $n_{[0]}=1$. For ${\varvec{n}},{\varvec{k}} \in \mathbb {Z}_+^m$, we define ${\varvec{n}}_{[{\varvec{k}}]}=\prod _{i=1}^m {n_i}_{[k_i]}$. For ${\varvec{i}},{\varvec{j}} \in \mathbb {Z}_+^m$, we write $\sum _{{\varvec{k}}={\varvec{i}}}^{{\varvec{j}}}$ for $\sum _{k_1=i_1}^{j_1}\sum _{k_2=i_2}^{j_2}\ldots \sum _{k_m=i_m}^{j_m}$. We take ${\varvec{0}}$ and ${\varvec{1}}$ to be vectors with all entries 0 and 1 respectively, with the dimensions being clear from the context. Finally, we let $f^{(r)}$ denote the *r*-th order derivative of the function *f*.

### B.1 Multitype single-household epidemic and key result

Consider a single-household SIR epidemic model with *m* types of individuals, labelled $1,2,\ldots ,m$. Suppose that, for $i=1,2,\ldots ,m$, there are initially $a_i$ infectives and $n_i$ susceptibles of type *i*, and let ${\varvec{a}}=(a_1,a_2,\ldots ,a_m)$ and ${\varvec{n}}=(n_1,n_2,\ldots ,n_m)$. For $i=1,2,\ldots ,m$, the infectious periods of type-*i* infectives are each distributed according to a random variable $I^{(i)}$. For $i,j=1,2,\ldots ,m$, the individual-to-individual infection rate from a given type-*i* infective to a given type-*j* susceptible is $\lambda _{ij}$. As in Sect. 2.1, such infections are governed by Poisson processes, and all Poisson processes and infectious periods are mutually independent.

To each type-*i* infective we attach a vector of *p* non-negative integer-valued random attributes distributed according to a vector random variable ${\varvec{A}}^{(i)} = (A_1^{(i)}, A_2^{(i)}, \ldots , A_p^{(i)})$. In our applications ${\varvec{A}}^{(i)}$ will describe the numbers of global infections of different types of individuals made by an infective in the single-household epidemic. The realisations of the random variables $(I^{(i)}, {\varvec{A}}^{(i)})$ are independent for distinct infectives and identically distributed for infectives of the same type. Note that $I^{(i)}$ and ${\varvec{A}}^{(i)}$ may be dependent. For $i=1,2,\ldots ,m$, let $T_i$ be the number of initial susceptibles of type *i* that are ultimately infected by the epidemic and let ${\varvec{A}}^{(i)} (T_i)$ be the sum of the attribute vectors over all $a_i + T_i$ infectives of type *i*. Further, let ${\varvec{T}}=(T_1,T_2,\ldots ,T_m)$ and let ${\varvec{A}}({\varvec{T}})=\sum _{i=1}^m {\varvec{A}}^{(i)} (T_i)$ be the sum of the attribute vectors over all $\sum _{i=1}^m (a_i+T_i)$ infectives in the epidemic. Thus, in applications, ${\varvec{A}}({\varvec{T}})$ will give the offspring random variable for the forward branching process. For ${\varvec{x}}=(x_1,x_2,\ldots ,x_m) \in \mathbb {R}^m$ and ${\varvec{s}}=(s_1,s_2,\ldots ,s_p) \in [0,1]^p$, let

$$\begin{aligned} \phi ({\varvec{x}},{\varvec{s}}) = E[{\varvec{x}}^{{\varvec{n}}-{\varvec{T}}} {\varvec{s}}^{{\varvec{A}}({\varvec{T}})}]. \end{aligned}$$

We give below an expression for $\phi ({\varvec{x}}, s)$ in terms of multivariate Gontcharoff polynomials, first studied by Lefèvre and Picard (1990), which we now define. Let ${\varvec{U}}=({\varvec{u}}_{{\varvec{j}}} \in \mathbb {R}^m :{\varvec{j}} \in \mathbb {Z}_+^m$) be a collection of real numbers. The Gontcharoff polynomials associated with ${\varvec{U}}$, denoted $(G_{{\varvec{k}}}({\varvec{x}} | {\varvec{U}}) ,\, {\varvec{k}}\in \mathbb {Z}_+^m , {\varvec{x}}\in \mathbb {R}^m)$, are defined recursively by

$$\begin{aligned} \sum _{{\varvec{j}}={\varvec{0}}}^{{\varvec{k}}} {\varvec{k}}_{[{\varvec{j}}]} {\varvec{u}}_{{\varvec{j}}}^{{\varvec{k}}-{\varvec{j}}} G_{{\varvec{j}}} ({\varvec{x}} | {\varvec{U}})={\varvec{x}}^{{\varvec{k}}} \quad ({\varvec{k}} \ge {\varvec{0}}). \end{aligned}$$

Note that $G_{{\varvec{0}}} ({\varvec{x}}|{\varvec{U}}) \equiv 1$ and that $G_{{\varvec{k}}} ({\varvec{x}}|{\varvec{U}})$ is a polynomial of degree $k_1,k_2,\ldots ,k_m$ in $x_1,x_2,\ldots ,x_m$, respectively, depending only on $({\varvec{u}}_{{\varvec{j}}} :{\varvec{j}} < {\varvec{k}})$. The following result is derived easily from Ball and O’Neill (1999, Theorem 5.1), so its proof is omitted.

#### Theorem 1

For $${\varvec{x}} \in \mathbb {R}^m$$ and $${\varvec{s}} \in [0,1]^p$$,

B.1 $$\begin{aligned} \phi ({\varvec{x}}, {\varvec{s}})=\sum _{{\varvec{j}}={\varvec{0}}}^{{\varvec{n}}} {\varvec{n}}_{[{\varvec{j}}]} ({\varvec{\psi }}({\varvec{s}},{\varvec{j}}))^{{\varvec{n}}+{\varvec{a}}-{\varvec{j}}} G_{{\varvec{j}}} ({\varvec{x}}|{\varvec{U}}), \end{aligned}$$

where $${\varvec{\psi }}({\varvec{s}},{\varvec{j}})=(\psi _1 ({\varvec{s}},{\varvec{j}}), \psi _2 ({\varvec{s}},{\varvec{j}}), \ldots , \psi _m ({\varvec{s}},{\varvec{j}}))$$, with

$$\begin{aligned} \psi _i ({\varvec{s}},{\varvec{j}}) = E \left[ \exp \left( -I^{(i)} \sum _{k=1}^m \lambda _{ik} j_k \right) {\varvec{s}}^{{\varvec{A}}^{(i)}} \right] \quad (i=1,2,\ldots ,m), \end{aligned}$$

and ${\varvec{U}} = ({\varvec{u}}_{{\varvec{j}}} :{\varvec{j}} \in \mathbb {Z}_+^m)$ has components ${\varvec{u}}_{{\varvec{j}}} = {\varvec{\psi }} ({\varvec{s}},{\varvec{j}})$.

We now describe how Theorem 1 can be used to determine offspring PGFs for the forward branching processes in the earlier parts of the paper. Recall that primary and secondary infectives in a household typically have distinct global degree distributions. Hence, in addition to being typed according to their vaccination status, individuals also need to be typed as primary or secondary. Thus we may write $m=m_P+m_S$, where types $1,2,\ldots ,m_P$ correspond to primary individuals and types $m_P +1, m_P +2, \ldots , m$ to secondary individuals. Write ${\varvec{a}}=({\varvec{a}}_P, {\varvec{a}}_S)$, ${\varvec{n}}=({\varvec{n}}_P, {\varvec{n}}_S)$, ${\varvec{x}}=({\varvec{x}}_P, {\varvec{x}}_S)$, ${\varvec{T}}=({\varvec{T}}_P, {\varvec{T}}_S)$ and ${\varvec{\psi }}({\varvec{s}},{\varvec{j}})=({\varvec{\psi }}_P ({\varvec{s}},{\varvec{j}}), {\varvec{\psi }}_S ({\varvec{s}},{\varvec{j}}))$ in the obvious fashion. Note that all susceptibles are secondary individuals, so ${\varvec{n}}_P={\varvec{0}}$, and all initial infectives are primary individuals, so ${\varvec{a}}_S={\varvec{0}}$. It follows that ${\varvec{T}}_P={\varvec{0}}$ and the index ${\varvec{j}}$ of the summation in (B.1) takes the form $({\varvec{0}},{\varvec{j}}_S)$. Let

$$\begin{aligned} {\tilde{\phi }} ({\varvec{x}}_S, {\varvec{s}}) = E\left[ {\varvec{x}}_S^{{\varvec{n}}_S - {\varvec{T}}_S} {\varvec{s}}^{{\varvec{A}}({\varvec{T}})} \right] \quad ({\varvec{x}}_S \in \mathbb {R}^{m_S}, \, {\varvec{s}}\in [0,1]^p ). \end{aligned}$$

Then the following corollary follows easily from Theorem 1. For $i=1,2,\ldots ,m$, let ${\varvec{\lambda }}_S^{(i)}=(\lambda _{i,m_P+1}, \lambda _{i,m_P+2}, \ldots , \lambda _{i,m})^{\top }$, where $\top$ denotes transpose.

#### Corollary 2

For $${\varvec{x}}_S \in \mathbb {R}^{m_S}$$ and $${\varvec{s}} \in [0,1]^p$$,

B.2 $$\begin{aligned} {\tilde{\phi }} ({\varvec{x}}_S, {\varvec{s}}) = \sum _{{\varvec{j}}_S={\varvec{0}}}^{{\varvec{n}}_S} {\varvec{n}}_{S_{[{\varvec{j}}_S]}} ({\tilde{{\varvec{\psi }}}}_P ({\varvec{s}},{\varvec{j}}_S))^{{\varvec{a}}_P} ({\tilde{{\varvec{\psi }}}}_S ({\varvec{s}},{\varvec{j}}_S))^{{\varvec{n}}_S-{\varvec{j}}_S} \, G_{{\varvec{j}}_S} ({\varvec{x}}_S | {\varvec{U}}_S), \end{aligned}$$

where ${\tilde{{\varvec{\psi }}}}_P ({\varvec{s}},{\varvec{j}}_S)=\left( {\tilde{\psi }}_1 ({\varvec{s}},{\varvec{j}}_S), {\tilde{\psi }}_2 ({\varvec{s}},{\varvec{j}}_S), \ldots , {\tilde{\psi }}_{m_P} ({\varvec{s}},{\varvec{j}}_S)\right)$ and ${\tilde{{\varvec{\psi }}}}_S ({\varvec{s}}, {\varvec{j}}_S) = \left( {\tilde{\psi }}_{m_P+1} ({\varvec{s}},{\varvec{j}}_S), {\tilde{\psi }}_{m_P+2} ({\varvec{s}},{\varvec{j}}_S), \ldots , {\tilde{\psi }}_m ({\varvec{s}},{\varvec{j}}_S)\right)$, with

B.3 $$\begin{aligned} {\tilde{\psi }}_i ({\varvec{s}},{\varvec{j}}_S)=E\left[ \exp (-I^{(i)} {\varvec{j}}_S {\varvec{\lambda }}_S^{(i)}) {\varvec{s}}^{{\varvec{A}}^{(i)}}\right] \quad (i=1,2,\ldots ,m) \end{aligned}$$

and ${\varvec{U}}_S = ( {\varvec{u}}_{{\varvec{j}}_S}^S :{\varvec{j}}_S \in \mathbb {Z}_+^{m_S})$ has components ${\varvec{u}}_{{\varvec{j}}_S}^S = {\tilde{{\varvec{\psi }}}}_S ({\varvec{s}},{\varvec{j}}_S)$.

We are primarily concerned with determining the PGF of ${\varvec{A}}({\varvec{T}})$, which of course is obtained by setting ${\varvec{x}}_S={\varvec{1}}$ in (B.2). Thus, all that remains is to determine ${\tilde{\psi }}_i ({\varvec{s}},{\varvec{j}}_S)$, which is application dependent. Note from (B.3) that it is sufficient to determine, for $\theta \in \mathbb {R}_+$ and ${\varvec{s}} \in [0,1]^p$,

$$\begin{aligned} {\hat{\psi }}_i (\theta ,{\varvec{s}}) = E\left[ \exp (-\theta I^{(i)}) {\varvec{s}} ^{{\varvec{A}}^{(i)}}\right] \quad (i=1,2,\ldots ,m), \end{aligned}$$

which we now do for the various models in the paper.

### B.2 No vaccination

This model is studied by Ball et al. (2010), so we just state the result. Note that there are two types of individual, primary (*P*) and secondary (*S*), with their number of uninfected global neighbours distributed according to $D_P \sim {\tilde{D}}-1$ and $D_S \sim D$, respectively. Further $p=1$, so ${\varvec{s}}$ is a scalar, *s* say. For a degree distribution *D* and real numbers $c_1,c_2,\theta$, define the function $F(D,c_1,c_2,\theta )$ by

B.4 $$\begin{aligned} F(D,c_1,c_2,\theta )=\sum _{r=0}^{\infty } \frac{c_1^r}{r!} \phi _I (\theta + r \lambda _G) f_D^{(r)} (c_2). \end{aligned}$$

Then, for $$A \in \{ P,S \}$$,

$$\begin{aligned} {\hat{\psi }}_A (\theta ,s) = F(D_A, 1-s, s, \theta ), \end{aligned}$$

cf. Ball et al. (2010, Theorem 1). Equation (B.4), in conjunction with Corollary 2, enables the PGFs $f_{C^{(n)}}$ and $f_{{\tilde{C}}^{(n)}}$, defined in Sect. 2.2.1, to be evaluated.

### B.3 Households based vaccination

Recall from Sect. 3 that with an all-or-nothing vaccine action all required PGFs and means can be expressed in terms of $f_{C^{(n)}}$, $f_{{\tilde{C}}^{(n)}}$ and $\mu _n (\lambda _L)$, so here we need consider only the non-random vaccine action model. This model has four types of individuals: primary-unvaccinated (*PU*), primary-vaccinated (*PV*), secondary-unvaccinated (*SU*) and secondary-vaccinated (*SV*), their number of uninfected global neighbours being distributed as $D_{PU}$, $D_{PV}$, $D_{SU}$ and $D_{SV}$, respectively. Recall from Sect. 3.3 that $D_{PU} \mathop {=}\limits ^{D} D_{PV} \sim {\tilde{D}}-1$ and $D_{SU} \mathop {=}\limits ^{D} D_{SV} \sim D$. For $F \in \{PU,PV,SU,SV\}$, the random attribute of interest is ${\varvec{A}}^{(F)}=(A_U^{(F)},A_V^{(F)})$, where $A_U^{(F)}$ and $A_V^{(F)}$ are the number of unvaccinated and vaccinated global infections made by a type-*F* infective in the single household epidemic. Thus $m_S=2$ and we determine ${\hat{\psi }}_F (\theta ,{\varvec{s}})$, where ${\varvec{s}}=(s_U,s_V)$.

Consider first ${\hat{\psi }}_{SU}(\theta ,{\varvec{s}})$. Conditioning on the degree $D_{SU}$ and infectious period *I* of a typical type-*SU* infective, $i^{*}$ say, yields

B.5 $$\begin{aligned} {\hat{\psi }}_{SU} (\theta ,{\varvec{s}}) = E_{D_{SU},I} \left[ \mathrm {e}^{-\theta I} E\left[ {\varvec{s}}^{{\varvec{A}}^{(SU)}} \mid D_{SU}, I\right] \right] . \end{aligned}$$

Let $N_V$ denote the number of $i^{*}$’s uninfected global neighbours that are vaccinated. Then $\left( A_U^{(SU)} \mid N_V, D_{SU}, I\right) \sim \text {Bin} \left( D_{SU} - N_V, 1-\mathrm {e}^{-\lambda _G I}\right)$ and independently $\left( A_V^{(SU)} \mid N_V, D_{SU}, I\right) \sim \text {Bin} \left( N_V, 1-\mathrm {e}^{-\lambda _G aI}\right)$, so

B.6 $$\begin{aligned}&E\left[ {\varvec{s}}^{{\varvec{A}}^{(SU)}} \mid N_V, D_{SU}, I\right] \nonumber \\&\quad =\left( \mathrm {e}^{-\lambda _G I} + (1-\mathrm {e}^{-\lambda _G I})s_U \right) ^{D_{SU}-N_V}\left( \mathrm {e}^{-\lambda _G aI} + (1-\mathrm {e}^{-\lambda _G aI})s_V\right) ^{N_V},\quad \quad \quad \quad \end{aligned}$$

whence, since $$(N_V \mid D_{SU}) \sim \text {Bin} (D_{SU},p_V)$$,

B.7 $$\begin{aligned} E\left[ {\varvec{s}}^{{\varvec{A}}^{(SU)}} \mid D_{SU}, I\right]= & {} \left( (1-p_V) \left[ \mathrm {e}^{-\lambda _G I} + (1- \mathrm {e}^{-\lambda _G I}) s_U \right] + p_V \left[ \mathrm {e}^{-\lambda _G aI} + (1-\mathrm {e}^{-\lambda _G aI})s_V \right] \right) ^{D_{SU}}. \nonumber \\ \end{aligned}$$

(As usual, $\text {Bin}(n,p)$ denotes a binomial distribution with parameters $n\in \mathbb {Z}_+$ and $p\in [0,1]$.) Let ${\hat{a}} ({\varvec{s}}) = p_V s_V + (1-p_V) s_U$, ${\hat{b}} ({\varvec{s}}) = p_V (1-s_V)$ and ${\hat{c}} ({\varvec{s}}) = (1-p_V)(1-s_U)$. Then, using (B.5) and (B.7),

B.8 $$\begin{aligned}&{\hat{\psi }}_{SU} (\theta ,{\varvec{s}}) =E_{D_{SU},I} \left[ \mathrm {e}^{-\theta I} \left( {\hat{a}} ({\varvec{s}}) + {\hat{b}} ({\varvec{s}}) \mathrm {e}^{-\lambda _G aI} + {\hat{c}} ({\varvec{s}}) \mathrm {e}^{-\lambda _G I}\right) ^{D_{SU}}\right] \nonumber \\&\quad =\sum _{k=0}^{\infty } P(D_{SU}=k) E_I \left[ \mathrm {e}^{-\theta I} \sum _{r=0}^k \sum _{l=0}^{k-r} \frac{k!}{r! l! (k-r-l)!} ({\hat{a}} ({\varvec{s}}))^{k-l-r} ({\hat{b}} ({\varvec{s}}) \mathrm {e}^{-\lambda _G aI})^r ({\hat{c}} ({\varvec{s}}) \mathrm {e}^{-\lambda _G I})^l \right] \nonumber \\&\quad =\sum _{r=0}^\infty \sum _{l=0}^\infty \sum _{k=r+l}^\infty P(D_{SU} = k) \frac{k!}{r! l! (k-r-l)!} ({\hat{a}} ({\varvec{s}}))^{k-l-r}({\hat{b}} ({\varvec{s}}))^r ({\hat{c}} ({\varvec{s}}))^l E\left[ \mathrm {e}^{-(\theta + (ar+l) \lambda _G)I}\right] . \end{aligned}$$

For a degree distribution *D* and real numbers $c_1,c_2,c_3,\theta ,a,b$, define the function $G(D,c_1,c_2,c_3,\theta ,a,b)$ by

B.9 $$\begin{aligned} G(D,c_1,c_2,c_3,\theta ,a,b)=\sum _{r=0}^\infty \sum _{l=0}^\infty \frac{c_2^r}{r!} \frac{c_3^l}{l!} \phi _I (\theta + \lambda _G b(ar+l)) f_D^{(r+l)} (c_1). \end{aligned}$$

Then (B.8) implies that

B.10 $$\begin{aligned} {\hat{\psi }}_{SU} (\theta ,{\varvec{s}})=G(D_{SU}, {\hat{a}}({\varvec{s}}),{\hat{b}}({\varvec{s}}),{\hat{c}}({\varvec{s}}),\theta ,a,1). \end{aligned}$$

A similar argument shows that

B.11 $$\begin{aligned} {\hat{\psi }}_{SV} (\theta ,{\varvec{s}}) = G(D_{SV}, {\hat{a}}({\varvec{s}}), {\hat{b}}({\varvec{s}}), {\hat{c}}({\varvec{s}}), \theta , a,b), \end{aligned}$$

and that ${\hat{\psi }}_{PU} (\theta ,{\varvec{s}})$ is given by (B.10), with $D_{SU}$ replaced by $D_{PU}$, and ${\hat{\psi }}_{PV} (\theta ,{\varvec{s}})$ is given by (B.11), with $D_{SV}$ replaced by $D_{PV}$.

If a closed-form expression for $f_D$ is available then $G(D,c_1,c_2,c_3,\theta ,a,b)$ may be computed by using a finite truncation of (B.9). If a closed-form expression for $f_D$ is unavailable then $G(D,c_1,c_2,c_3,\theta ,a,b)$ may be computed by using a finite truncation of a corresponding triple sum (cf. (B.8)).

Note that if the infectious period is constant, say $I \equiv \iota$, then (B.5) to (B.7) imply that

$$\begin{aligned} {\hat{\psi }}_{SU} (\theta ,{\varvec{s}}) = \mathrm {e}^{-\theta \iota } f_{D_{SU}} \left( {\hat{a}}({\varvec{s}}) + \mathrm {e}^{-\lambda _G a \iota } {\hat{b}}({\varvec{s}}) + \mathrm {e}^{-\lambda _G \iota } {\hat{c}}({\varvec{s}})\right) \end{aligned}$$

and corresponding expressions for ${\hat{\psi }}_{SV} (\theta ,{\varvec{s}})$, ${\hat{\psi }}_{PU} (\theta ,{\varvec{s}})$ and ${\hat{\psi }}_{PV} (\theta ,{\varvec{s}})$ are easily derived.

### B.4 Acquaintance vaccination

This model has eight types of individuals, six primary and two secondary, which, as in Sect. 4.2, we label $1,2,\ldots ,6$ and *U*, *V*, respectively. For each type *i*, the random attribute of interest ${\varvec{A}}^{(i)}=(A^{(i)}_1,A^{(i)}_2,\ldots ,A^{(i)}_6)$, where $A^{(i)}_j$ is the number of global infections of a type *j* individual made by an infective type *i* individual. Thus we determine, in an obvious notation, ${\hat{\psi }}_i(\theta ,{\varvec{s}})$ ($i=1,2,\ldots ,6$), ${\hat{\psi }}_U (\theta ,{\varvec{s}})$ and ${\hat{\psi }}_V (\theta ,{\varvec{s}})$; for $\theta \in \mathbb {R}_+$ and ${\varvec{s}}=(s_1,s_2,\ldots ,s_6) \in [0,1]^6$. It is convenient to treat the all-or-nothing and non-random vaccine action models separately.

#### B.4.1 All-or-nothing vaccine action

For $i=1,2,\ldots ,6$, recall that ${\tilde{D}}(i)$ is the global degree of a typical type-*i* primary individual, $i^{*}$ say, and also that ${\tilde{{\varvec{X}}}}_i = ({\tilde{X}}_{i1}, {\tilde{X}}_{i2}, \ldots , {\tilde{X}}_{i6})$, where ${\tilde{X}}_{ij}$ is the number of $i^{*}$’s next-generation neighbours that have type *j*. Recall that types 3 and 6 have not been vaccinated and types 1, 2, 4 and 5 have been vaccinated. Thus

$$\begin{aligned} \left( A_j^{(i)}| {\tilde{X}}_{ij},I\right) \sim \left\{ \begin{array}{ll}{\text {Bin}}\left( {\tilde{X}}_{ij}, 1-\mathrm {e}^{-\lambda _GI}\right) &{}\quad \text {if }j=3,6,\\ {\text {Bin}}\left( {\tilde{X}}_{ij}, (1-\varepsilon )(1-\mathrm {e}^{-\lambda _GI})\right) &{}\quad \text {if } j=1,2,4,5, \end{array} \right. \end{aligned}$$

and

B.12 $$\begin{aligned} E\left[ {\varvec{s}}^{{\varvec{A}}^{(i)}} | {\tilde{{\varvec{X}}}}_i,I\right] =\prod _{j=3,6}&\left( \mathrm {e}^{-\lambda _GI} + \left. (1-\mathrm {e}^{-\lambda _GI})s_j\right) ^{{\tilde{X}}_{ij}}\right. \nonumber \\&\times \prod _{j=1,2,4,5} \left( \varepsilon + (1- \varepsilon ) \mathrm {e}^{-\lambda _GI} + (1-\varepsilon )(1-\mathrm {e}^{-\lambda _G I} ) s_j\right) ^{{\tilde{X}}_{ij}}. \end{aligned}$$

Further, for $i=1,3,4,6$, the types of the ${\tilde{D}}(i)-1$ of $i^{*}$’s next-generation neighbours are chosen independently according to ${\hat{p}}_{ij}$, defined in Sect. A.1.2, so

B.13 $$\begin{aligned} E\left[ {\varvec{s}}^{{\varvec{A}}^{(i)}} | {\tilde{D}}(i),I\right] = \left( {\hat{p}}_i ({\varvec{s}},\varepsilon ) + (1-{\hat{p}}_i ({\varvec{s}},\varepsilon )) \mathrm {e}^{-\lambda _GI}\right) ^{{\tilde{D}}(i)-1}, \end{aligned}$$

where

$$\begin{aligned} {\hat{p}}_i ({\varvec{s}},\varepsilon ) = \sum _{j=3,6} {\hat{p}}_{ij} s_j + \sum _{j=1,2,4,5} {\hat{p}}_{ij} (\varepsilon + (1-\varepsilon ) s_j). \end{aligned}$$

Hence,

B.14 $$\begin{aligned} {\hat{\psi }}_i (\theta ,{\varvec{s}})&=E\left[ \mathrm {e}^{-\theta I} {\varvec{s}}^{{\varvec{A}}^{(i)}}\right] \nonumber \\&=E_I \left[ \mathrm {e}^{-\theta I} E_{{\tilde{D}}(i)} \left[ E\left[ {\varvec{s}}^{{\varvec{A}}^{(i)}} | {\tilde{D}}(i),I \right] \right] \right] \nonumber \\&=E_I \left[ \mathrm {e}^{-\theta I} E_{{\tilde{D}}(i)} \left[ ( {\hat{p}}_i ({\varvec{s}},\varepsilon ) + (1-{\hat{p}}_i ({\varvec{s}},\varepsilon )) \mathrm {e}^{-\lambda _GI})^{{\tilde{D}}(i)-1} \right] \right] . \end{aligned}$$

A similar argument to the derivation of (B.8), using the binomial theorem rather than the multinomial theorem, then yields

$$\begin{aligned} {\hat{\psi }}_i (\theta ,{\varvec{s}}) = F({\tilde{D}}(i)-1, 1-{\hat{p}}_i ({\varvec{s}},\varepsilon ), {\hat{p}}_i ({\varvec{s}},\varepsilon ), \theta ). \end{aligned}$$

For $i=2$ and $i=5$, (B.12) still holds but $f_{{\tilde{{\varvec{X}}}}_i} ({\varvec{s}})$ is given by (A.9) and (A.10), respectively. Omitting the details, similar arguments to the above show that, for $i=2,5$,

B.15 $$\begin{aligned} {\hat{\psi }}_i (\theta ,{\varvec{s}})= & {} {\tilde{p}}_V^{-1} \left[ F\left( {\tilde{D}}-1,{\tilde{p}}_i ({\varvec{s}},0), {\tilde{q}}_i ({\varvec{s}},0),\theta \right) - F\left( {\tilde{D}}-1, {\tilde{p}}_i ({\varvec{s}},p_N), {\tilde{q}}_i ({\varvec{s}}, p_N ), \theta \right) \right] , \nonumber \\ \end{aligned}$$

where ${\tilde{p}}_i({\varvec{s}},x) = p_S(1-x){\tilde{p}}_{i1}({\varvec{s}}) + (1-p_S){\tilde{p}}_{i2}({\varvec{s}})$ and ${\tilde{q}}_i({\varvec{s}},x) = 1-p_Sx-{\tilde{p}}_i({\varvec{s}},x)$, with

$$\begin{aligned} {\tilde{p}}_{21}({\varvec{s}})&= p_N(1-\varepsilon )(1-s_1) + (1-p_N)[{\tilde{p}}_V(1-\varepsilon )(1-s_2) + (1-{\tilde{p}}_V)(1-s_3)], \\ {\tilde{p}}_{22}({\varvec{s}})&= p_N(1-\varepsilon )(1-s_4) + (1-p_N)[{\tilde{p}}_V(1-\varepsilon )(1-s_5) + (1-{\tilde{p}}_V)(1-s_6)], \\ {\tilde{p}}_{51}({\varvec{s}})&= {\tilde{p}}_V(1-\varepsilon )(1-s_2) + (1-{\tilde{p}}_V)(1-s_3) \quad \text{ and } \\ {\tilde{p}}_{52}({\varvec{s}})&= {\tilde{p}}_V(1-\varepsilon )(1-s_5) + (1-{\tilde{p}}_V)(1-s_6). \end{aligned}$$

To simplify the exposition we write (B.15) as

$$\begin{aligned} {\hat{\psi }}_i (\theta ,{\varvec{s}}) = {\tilde{p}}_V^{-1} \Delta _{0,p_N} F\left( {\tilde{D}}-1,{\tilde{p}}_i ({\varvec{s}},x), {\tilde{q}}_i ({\varvec{s}},x),\theta \right) . \end{aligned}$$

In the sequel we use, without comment, a similar notation for differences of other functions evaluated at 0 and $p_N$.

Turning now to secondary individuals, note that a type-*U* individual has global degree and therefore number of next-generation neighbours distributed as $D_U$. Such an individual is sampled with probability $p_S$, in which case the type of each next-generation neighbour is distributed according to ${\hat{p}}_{3j}$, otherwise it is unsampled and the type of each next-generation neighbour is distributed according to ${\hat{p}}_{6j}$. Thus

B.16 $$\begin{aligned} {\hat{\psi }}_U (\theta ,{\varvec{s}})= & {} p_S F\left( D_U,1-{\hat{p}}_3 ({\varvec{s}},\varepsilon ), {\hat{p}}_3 ({\varvec{s}},\varepsilon ),\theta \right) +(1-p_S)F\left( D_U,1-{\hat{p}}_6 ({\varvec{s}},\varepsilon ),{\hat{p}}_6 ({\varvec{s}},\varepsilon ),\theta \right) .\nonumber \\ \end{aligned}$$

Similarly,

B.17 $$\begin{aligned} {\hat{\psi }}_V (\theta ,{\varvec{s}})= p_V^{-1} \left[ p_S \Delta _{0,p_N} F\left( D_V, {\tilde{p}}_2 ({\varvec{s}},x), {\tilde{q}}_2 ({\varvec{s}},x),\theta \right) +\, (1-p_S) \Delta _{0,p_N} F\left( D_V,{\tilde{p}}_5 ({\varvec{s}},x), {\tilde{q}}_5 ({\varvec{s}},x),\theta \right) \right] . \end{aligned}$$

To simplify the exposition we write (B.16) and (B.17) as

$$\begin{aligned} {\hat{\psi }}_U (\theta ,{\varvec{s}})=p_S^{3,6} F(D_U,1-{\hat{p}}({\varvec{s}},\varepsilon ),{\hat{p}}({\varvec{s}},\varepsilon ),\theta ) \end{aligned}$$

and

$$\begin{aligned} {\hat{\psi }}_V (\theta ,{\varvec{s}}) = p_V^{-1} p_S^{2,5} \Delta _{0,p_N} F(D_V,{\tilde{p}}({\varvec{s}},x), {\tilde{q}}({\varvec{s}},x),\theta ). \end{aligned}$$

In the sequel, we use a similar notation without comment.

The above expressions simplify appreciably if the infectious period is constant, say $I \equiv \iota$. In that case, Eq. (B.14) implies that

$$\begin{aligned} {\hat{\psi }}_i (\theta ,{\varvec{s}}) = \mathrm {e}^{-\theta \iota } f_{{\tilde{D}}(i)-1} \left( {\hat{p}}_i ({\varvec{s}},\varepsilon ) + (1-{\hat{p}}_i ({\varvec{s}},\varepsilon )) \mathrm {e}^{-\lambda _G \iota }\right) \quad (i=1,3,4,6) \end{aligned}$$

and exploiting the PGFs for ${\tilde{{\varvec{X}}}}_2$ and ${\tilde{{\varvec{X}}}}_5$ [see (A.9) and (A.10)] yields

$$\begin{aligned} {\hat{\psi }}_i (\theta ,{\varvec{s}}) = {\tilde{p}}_V^{-1} \mathrm {e}^{-\theta \iota } \Delta _{0,p_N} f_{{\tilde{D}}-1} \left( {\tilde{q}}_i ({\varvec{s}},x) + {\tilde{p}}_i ({\varvec{s}},x) \mathrm {e}^{-\lambda _G \iota }\right) \quad (i=2,5). \end{aligned}$$

Similar arguments show that

$$\begin{aligned} {\hat{\psi }}_U (\theta ,{\varvec{s}})= \mathrm {e}^{-\theta \iota } p_S^{3,6} f_{D_U} \left( {\hat{p}} ({\varvec{s}},\varepsilon ) + (1-{\hat{p}} ({\varvec{s}},\varepsilon )) \mathrm {e}^{-\lambda _G \iota }\right) \end{aligned}$$

and

$$\begin{aligned} {\hat{\psi }}_V (\theta ,{\varvec{s}}) = p_V^{-1} \mathrm {e}^{-\theta \iota } p_S^{2,5} \Delta _{0,p_N} f_{D_V} \left( {\tilde{q}} ({\varvec{s}},x) + {\tilde{p}} ({\varvec{s}},x) \mathrm {e}^{-\lambda _G \iota } \right) . \end{aligned}$$

#### B.4.2 Non-random vaccine

Note that now

B.18 $$\begin{aligned} (A_j^{(i)} | {\tilde{X}}_{ij},I) \sim \left\{ \begin{array}{ll} {\text {Bin}} ( {\tilde{X}}_{ij}, 1-\mathrm {e}^{-\lambda _G I})&{}\quad \text {if } i,j \in \{3,6\},\\ {\text {Bin}} ({\tilde{X}}_{ij}, 1-\mathrm {e}^{-a \lambda _G I} )&{}\quad \text {if } i \in \{3,6\}, ~ j \in \{ 1,2,4,5\},\\ {\text {Bin}} ( {\tilde{X}}_{ij}, 1-\mathrm {e}^{-b \lambda _G I})&{}\quad \text {if } i \in \{ 1,2,4,5\}, ~ j \in \{ 3,6 \},\\ {\text {Bin}} ( {\tilde{X}}_{ij}, 1-\mathrm {e}^{-ab \lambda _G I})&{}\quad \text {if } i,j \in \{ 1,2,4,5 \}. \end{array} \right. \end{aligned}$$

For $$i \in \{1, 3,4,6 \}$$, let

$$\begin{aligned} {\hat{a}}_i ({\varvec{s}}) = \sum _{j=1}^6 {\hat{p}}_{ij} s_j, \quad {\hat{b}}_i ({\varvec{s}}) = \sum _{j=1,2,4,5} {\hat{p}}_{ij} (1-s_j) \quad \text {and} \quad {\hat{c}}_i ({\varvec{s}}) = \sum _{j=3,6} {\hat{p}}_{ij} (1-s_j). \end{aligned}$$

Suppose that $i \in \{ 3,6 \}$. Then, arguing as in the derivation of (B.13),

$$\begin{aligned} E\left[ {\varvec{s}}^{{\varvec{A}}^{(i)}} | {\tilde{D}}(i),I\right] = \left( {\hat{a}}_i ({\varvec{s}}) + {\hat{b}}_i ({\varvec{s}}) \mathrm {e}^{-a \lambda _G I} + {\hat{c}}_i ({\varvec{s}}) \mathrm {e}^{-\lambda _G I}\right) ^{{\tilde{D}}(i)-1} , \end{aligned}$$

whence

B.19 $$\begin{aligned} {\hat{\psi }}_i (\theta ,{\varvec{s}}) = E_I \left[ \mathrm {e}^{- \theta I} E_{{\tilde{D}}(i)} \left[ \left( {\hat{a}}_i ({\varvec{s}}) + {\hat{b}}_i ({\varvec{s}}) \mathrm {e}^{-a \lambda _G I} + {\hat{c}}_i ({\varvec{s}}) \mathrm {e}^{-\lambda _G I} \right) ^{{\tilde{D}}(i)-1} \right] \right] . \end{aligned}$$

Arguing as in the derivation of (B.8) then yields that

$$\begin{aligned} {\hat{\psi }}_i (\theta ,{\varvec{s}}) = G\left( {\tilde{D}}(i)-1, {\hat{a}}_i ({\varvec{s}}), {\hat{b}}_i ({\varvec{s}}), {\hat{c}}_i ({\varvec{s}}), \theta , a, 1\right) . \end{aligned}$$

Similarly, for $$i \in \{ 1,4 \}$$,

$$\begin{aligned} {\hat{\psi }}_i (\theta ,{\varvec{s}}) = G\left( {\tilde{D}}(i) -1, {\hat{a}}_i ({\varvec{s}}), {\hat{b}}_i ({\varvec{s}}), {\hat{c}}_i ({\varvec{s}}), \theta , a,b\right) . \end{aligned}$$

Suppose that $i=5$. Then, using (B.18),

$$\begin{aligned} E\left[ {\varvec{s}}^{{\varvec{A}}^{(5)}} | {\tilde{{\varvec{X}}}}_5, I\right]= & {} \prod _{j=3,6} \left( \mathrm {e}^{-b \lambda _G I} + (1-\mathrm {e}^{-b \lambda _GI})s_j\right) ^{{\tilde{X}}_{5j}}\\&\times \prod _{j=1,2,4,5} \left( \mathrm {e}^{-ab \lambda _G I} + (1-\mathrm {e}^{-ab \lambda _G I} ) s_j \right) ^{{\tilde{X}}_{5j}}. \end{aligned}$$

Invoking (A.10) now gives

B.20 $$\begin{aligned} {\hat{\psi }}_5 (\theta ,{\varvec{s}}) = {\tilde{p}}_V^{-1} E\left[ \mathrm {e}^{-\theta I} \Delta _{0,p_N} f_{{\tilde{D}}-1} \left( {\hat{g}}_5 ({\varvec{h}}_5^F ({\varvec{s}},I), x)\right) \right] , \end{aligned}$$

where ${\varvec{h}}_5^F ({\varvec{s}},I) = (h_{51}^F (s_1,I), h_{52}^F (s_2,I), \ldots , h_{56}^F (s_6,I))$, with $h_{5j}^F(s,I)=\mathrm {e}^{-b \lambda _G I} + (1-\mathrm {e}^{-b \lambda _G I})s$ if $j=3,6$ and $h_{5j}^F (s,I) = \mathrm {e}^{-ab \lambda _G I} + (1-\mathrm {e}^{-ab \lambda _G I})s$ if $j=1,2,4,5$; and ${\hat{g}}_5({\varvec{s}},x)$ is as defined immediately following (A.10) in Sect. A.1.2. A simple calculation now shows that

B.21 $$\begin{aligned} {\hat{g}}_5 \left( {\varvec{h}}_5^F ({\varvec{s}},I), x \right) = {\hat{a}}_5 ({\varvec{s}},x) + {\hat{b}}_5 ({\varvec{s}},x) \mathrm {e}^{-ab \lambda _G I} + {\hat{c}}_5 ({\varvec{s}},x) \mathrm {e}^{-b \lambda _G I}, \end{aligned}$$

where ${\hat{a}}_5 ({\varvec{s}},x)=p_S(1-x)[{\tilde{p}}_V s_2 + (1-{\tilde{p}}_V)s_3] + (1-p_S)[{\tilde{p}}_V s_5 + (1-{\tilde{p}}_V)s_6]$, ${\hat{b}}_5 ({\varvec{s}},x)=p_S(1-x) {\tilde{p}}_V (1-s_2) + (1-p_S) {\tilde{p}}_V (1-s_5)$ and lastly ${\hat{c}}_5 ({\varvec{s}},x)=p_S(1-x)(1-{\tilde{p}}_V)(1-s_3) + (1-p_S)(1-{\tilde{p}}_V)(1-s_6)$. Arguing as in the derivation of (B.8) now gives, for $i=5$,

B.22 $$\begin{aligned} {\hat{\psi }}_i (\theta ,{\varvec{s}}) = {\tilde{p}}_V^{-1} \Delta _{0,p_N} G\left( {\tilde{D}}-1, {\hat{a}}_i ({\varvec{s}},x),{\hat{b}}_i ({\varvec{s}},x), {\hat{c}}_i ({\varvec{s}},x), \theta , a,b\right) . \end{aligned}$$

A similar argument shows that (B.22) holds also for $i=2$, with

$$\begin{aligned} {\hat{a}}_2 ({\varvec{s}},x)&=p_S(1-x) \{ p_N s_1 + (1-p_N) [ {\tilde{p}}_V s_2 + (1-{\tilde{p}}_V) s_3]\}\\&\qquad + (1-p_S) \{ p_N s_4 + (1-p_N) [ {\tilde{p}}_V s_5 + (1-{\tilde{p}}_V) s_6 ] \},\\ {\hat{b}}_2 ({\varvec{s}},x)&= p_S (1-x) [p_N (1-s_1) + (1-p_N) {\tilde{p}}_V (1-s_2)]\\&\qquad + (1-p_S) [p_N (1-s_4) + (1-p_N) {\tilde{p}}_V (1-s_5)] \text{ and }\\ {\hat{c}}_2 ({\varvec{s}},x)&= (1-p_N)(1-{\tilde{p}}_V) [p_S (1-x)(1-s_3) + (1-p_S)(1-s_6)]. \end{aligned}$$

Expressions for ${\hat{\psi }}_U (\theta ,{\varvec{s}})$ and ${\hat{\psi }}_V (\theta ,{\varvec{s}})$ are derived in exactly the same way as with an all-or-nothing vaccine, yielding

$$\begin{aligned} {\hat{\psi }}_U (\theta ,{\varvec{s}})=p_S^{3,6} G\left( D_U , {\hat{a}} ({\varvec{s}}), {\hat{b}} ({\varvec{s}}), {\hat{c}} ({\varvec{s}}), \theta , a, 1\right) \end{aligned}$$

and

$$\begin{aligned} {\hat{\psi }}_V (\theta ,{\varvec{s}}) = p_V^{-1} p_S^{2,5} \Delta _{0,p_N} G\left( D_V, {\hat{a}}({\varvec{s}},x), {\hat{b}} ({\varvec{s}},x), {\hat{c}}({\varvec{s}},x),\theta ,a,b\right) . \end{aligned}$$

As usual, the above expressions simplify if $I \equiv \iota$. Eq. (B.19) then implies that, for $i=3,6$,

$$\begin{aligned} {\hat{\psi }}_i (\theta ,{\varvec{s}}) = \mathrm {e}^{-\theta \iota } f_{{\tilde{D}}(i)} \left( {\hat{a}}_i ({\varvec{s}}) + {\hat{b}}_i ({\varvec{s}}) \mathrm {e}^{-a \lambda _G \iota } + {\hat{c}}_i ({\varvec{s}}) \mathrm {e}^{-\lambda _G \iota }\right) , \end{aligned}$$

which also holds for $i=1,4$, provided $\lambda _G$ is replaced by $b \lambda _G$. Further, exploiting (B.20), (B.21) and similar equations for ${\hat{\psi }}_2 (\theta ,{\varvec{s}})$, gives, for $i=2,5$,

$$\begin{aligned} {\hat{\psi }}_i (\theta ,{\varvec{s}}) = {\tilde{p}}_V^{-1} \mathrm {e}^{-\theta \iota } \Delta _{0,p_N} f_{{\tilde{D}}-1} \left( {\hat{a}}_i ({\varvec{s}},x) + {\hat{b}}_i ({\varvec{s}},x) \mathrm {e}^{-ab \lambda _G \iota } + {\hat{c}}_i ({\varvec{s}},x) \mathrm {e}^{-b \lambda _G \iota }\right) . \end{aligned}$$

Lastly, we also find that

$$\begin{aligned} {\hat{\psi }}_U (\theta ,{\varvec{s}}) = \mathrm {e}^{-\theta \iota } p_S^{3,6} f_{D_U} \left( {\hat{a}}({\varvec{s}}) + {\hat{b}}({\varvec{s}}) \mathrm {e}^{-a \lambda _G \iota } + {\hat{c}}({\varvec{s}}) \mathrm {e}^{-\lambda _G \iota }\right) \end{aligned}$$

and

$$\begin{aligned} {\hat{\psi }}_V (\theta ,s) = p_V^{-1} \mathrm {e}^{-\theta \iota } p_S^{2,5} \Delta _{0,p_N} f_{D_V} \left( {\hat{a}}({\varvec{s}},x) + {\hat{b}}({\varvec{s}},x) \mathrm {e}^{-ab \lambda _G \iota } + {\hat{c}} ({\varvec{s}},x) \mathrm {e}^{-b \lambda _G \iota }\right) . \end{aligned}$$

### B.5 Local susceptibility set size

In this section we give formulae for the probability mass functions of the size of local susceptibility sets. The corresponding PGFs are then simple to calculate numerically. Consider first the model without vaccination and recall the definition of $M^{(n)}$ in Sect. 2.2.2. Then it follows directly from Ball (2000, Lemma 3.1) (see also Ball and Neal (2002, Lemma 3.1), which gives the same result but not in terms of Gontcharoff polynomials) that

B.23 $$\begin{aligned} P(M^{(n)} = k) = (n-1)_{[k]} q_{k+1}^{n-1-k} G_{k+1} (1|V) \quad (k=0,1,\ldots ,n-1), \end{aligned}$$

where $$q_i = \phi _I (\lambda _L i)$$ and $$V=(q_{i+1} :i=0,1,\ldots )$$.

Consider next the model with a non-random vaccine action and recall the definition of ${\varvec{M}}_A^{(n,v)} = (M_{AU}^{(n,v)}, M_{AV}^{(n,v)})$ ($A \in \{ U,V \}$) in Sect. 3.3. It is convenient to now use a different notation. For ${\varvec{n}}=(n_U,n_V) \in \mathbb {Z}_+^2$, let ${\hat{{\varvec{M}}}}_U^{({\varvec{n}})}=({\hat{M}}_{UU}^{({\varvec{n}})}, {\hat{M}}_{UV}^{({\varvec{n}})})$, where ${\hat{M}}_{UU}^{({\varvec{n}})}$ and ${\hat{M}}_{UV}^{({\varvec{n}})}$ are the numbers of unvaccinated and vaccinated individuals, respectively, in the local susceptibility set of an unvaccinated individual, $i^{*}$ say, who resides in a household containing $n_U$ other unvaccinated individuals and $n_V$ vaccinated individuals, so the household has size $n_U + n_V + 1$. Note that ${\hat{M}}_{UU}^{({\varvec{n}})}$ does *not* include the individual $i^{*}$. Define ${\hat{{\varvec{M}}}}_V^{({\varvec{n}})} = ({\hat{M}}_{VU}^{({\varvec{n}})}, {\hat{M}}_{VV}^{({\varvec{n}})})$ similarly, but for a vaccinated individual. Hence, to connect with the notation in Sect. 3.3, ${\varvec{M}}_U^{(n,v)}={\hat{{\varvec{M}}}}_U^{(n-1-v,v)}$ and ${\varvec{M}}_V^{(n,v)}={\hat{{\varvec{M}}}}_V^{(n-v,v-1)}$. For ${\varvec{n}} \ge {\varvec{0}}$ and $A \in \{ U,V \}$, the probability mass function of ${\hat{{\varvec{M}}}}_A^{({\varvec{n}})}$ can be derived using the same argument as in the proof of Lemma 3.1 in Ball (2000). For brevity, we omit the details and just state the result.

For ${\varvec{j}}=(j_U,j_V) \ge {\varvec{0}}$, let ${\varvec{q}}_{{\varvec{j}}} = (q_{{\varvec{j}}}^U, q_{{\varvec{j}}}^V)$, where $q_{{\varvec{j}}}^U = \phi _I (\lambda _L (j_U+bj_V))$ and $q_{{\varvec{j}}}^V = \phi _I (a\lambda _L (j_U+bj_V))$. Let ${\varvec{1}}_U = (1,0)$ and ${\varvec{1}}_V=(0,1)$. Then, for ${\varvec{n}} \ge {\varvec{0}}$ and $A \in \{ U,V \}$,

B.24 $$\begin{aligned} P\left( {\hat{{\varvec{M}}}}_A^{({\varvec{n}})} = {\varvec{k}}\right) = {\varvec{n}}_{[{\varvec{k}}]} {\varvec{q}}_{{\varvec{k}}+{\varvec{1}}_A}^{{\varvec{n}}-{\varvec{k}}} G_{{\varvec{k}}} ({\varvec{1}} | {\varvec{V}}^A) \quad ({\varvec{0}} \le {\varvec{k}} \le {\varvec{n}}-{\varvec{1}}_A), \end{aligned}$$

where ${\varvec{V}}^A = ({\varvec{v}}_{{\varvec{j}}}^A :{\varvec{j}} \ge {\varvec{0}})$ has components ${\varvec{v}}_{{\varvec{j}}}^A = {\varvec{q}}_{{\varvec{j}}+{\varvec{1}}_A}^A$.

In the model with an all-or-nothing vaccine action we can calculate the mass function of ${\varvec{M}}_A^{(n,v)}$ by conditioning on the number, $v_S$ say, of the *v* vaccinations that are successful. The mass function of ${\varvec{M}}_A^{(n-v_S,v-v_S)}$ can then be calculated as above, but with $q_{{\varvec{j}}}^U = q_{{\varvec{j}}}^V = \phi _I (\lambda _L (j_U+j_V))$.

### B.6 Mean local epidemic size

The mean single-household epidemic final size $\mu _n (\lambda _L)$, defined in Sect. 2.2.1, is given in terms of Gontcharoff polynomials by

B.25 $$\begin{aligned} \mu _n (\lambda _L) = n-1- \sum _{i=1}^{n-1} (n-1)_{[i]} q_i^{n-i} G_{i-1} (1|V), \end{aligned}$$

where $q_i= \phi _I (i \lambda _L )$ and $V=(q_{i+1} :i=0,1,\ldots )$; cf. Lefévre and Picard (1990, Corollary 3.3).

Finally, we consider the means $\mu ^{(n,v)} (A,A')$ ($A,A' \in \{ U,V \}$) defined in Sect. 3.3. Again it is convenient to use a different notation. For ${\varvec{n}}=(n_U,n_V) \ge {\varvec{0}}$, let ${\hat{\mu }}^{({\varvec{n}})} (U,U)$ and ${\hat{\mu }}^{({\varvec{n}})} (U,V)$ denote respectively the mean number of unvaccinated and vaccinated susceptibles that are ultimately infected in a single-household epidemic with 1 initial infective, who is unvaccinated, $n_U$ unvaccinated susceptibles and $n_V$ vaccinated susceptibles. Define ${\hat{\mu }}^{({\varvec{n}})} (V,U)$ and ${\hat{\mu }}^{({\varvec{n}})} (V,V)$ similarly, for when the initial infective is vaccinated. Thus, to connect with the notation in Sect. 3.3, for $A \in \{ U,V \}$, $\mu ^{(n,v)} (U,A)={\hat{\mu }}^{(n-1-v,v)} (U,A)$ and $\mu ^{(n,v)} (V,A) = {\hat{\mu }}^{(n-v,v-1)} (V,A)$. Suitable differentiation applied to Theorem 3.5 of Ball (1986), or Corollary 4.4 of Picard and Lefèvre (1990), yields that, for ${\varvec{n}} \ge 0$ and $A,A' \in \{ U,V \}$,

$$\begin{aligned} {\hat{\mu }}^{({\varvec{n}})} (A,A') = n_{A'} - \sum _{{\varvec{k}}={\varvec{1}}_{A'}}^{{\varvec{n}}} {\varvec{n}}_{[{\varvec{k}}]} {\varvec{q}}_{{\varvec{k}}}^{{\varvec{n}}+{\varvec{1}}_A - {\varvec{k}}} G_{{\varvec{k}}-{\varvec{1}}_{A'}} ({\varvec{1}} | {\varvec{V}}^{A'}), \end{aligned}$$

where ${\varvec{q}}_{{\varvec{k}}}$ (${\varvec{k}} \ge {\varvec{0}}$) and ${\varvec{V}}^A$ ($A \in \{ U,V \}$) are as in (B.24).
